# White light emission from a single organic molecule with dual phosphorescence at room temperature

**DOI:** 10.1038/s41467-017-00362-5

**Published:** 2017-09-04

**Authors:** Zikai He, Weijun Zhao, Jacky W. Y. Lam, Qian Peng, Huili Ma, Guodong Liang, Zhigang Shuai, Ben Zhong Tang

**Affiliations:** 1 Department of Chemistry, Division of Life Science, Institute of Molecular Functional Materials, Division of Biomedical Engineering, State Key Laboratory of Molecular Neuroscience, Institute for Advanced Study, and Hong Kong Branch of Chinese National Engineering Research Center for Tissue Restoration and Reconstruction, The Hong Kong University of Science and Technology, Clear Water Bay, Kowloon, Hong Kong, China; 2 School of Science, Harbin Institute of Technology Shenzhen, HIT Campus of University Town of Shenzhen, Shenzhen, 518055 China; 3 HKUST Shenzhen Research Institute, No. 9 Yuexing 1st RD, South Area, Hi-tech Park Nanshan, Shenzhen, 518000 China; 4 Key Laboratory of Organic Solids, Beijing National Laboratory for Molecular Science, Institute of Chemistry, Chinese Academy of Sciences, Beijing, 100190 China; 5 Key Laboratory of Organic OptoElectronics and Molecular Engineering, Department of Chemistry, Tsinghua University, Beijing, 100084 China; 6 DSAP, PCFM and GDHPPC Lab, School of Materials Science and Engineering, School of Chemistry and Chemical Engineering, Sun Yat-Sen University, Guangzhou, 510275 China

## Abstract

The development of single molecule white light emitters is extremely challenging for pure phosphorescent metal-free system at room temperature. Here we report a single pure organic phosphor, namely 4-chlorobenzoyldibenzothiophene, emitting white room temperature phosphorescence with Commission Internationale de l’Éclair-age coordinates of (0.33, 0.35). Experimental and theoretical investigations reveal that the white light emission is emerged from dual phosphorescence, which emit from the first and second excited triplet states. We also demonstrate the validity of the strategy to achieve metal-free pure phosphorescent single molecule white light emitters by intrasystem mixing dual room temperature phosphorescence arising from the low- and high-lying triplet states.

## Abstract (web summary)

The development of single molecule white light-emitters is extremely challenging for pure phosphorescent metal-free systems at room temperature. Here the authors show a single pure organic room temperature phosphor, 4-chlorobenzoyldibenzothiophene, utilizing the emission from both T_1_ and T_2_ states.

## Introduction

White organic light-emitting materials, devices, and processes have attracted continuous attention for their fundamental importance and practical implication^1–6^. Most examples reported so far rely on a combination of multi-components with emission color covering the entire visible range. Compared to these combined emitters, single molecule white light emitters (SMWLEs) are expected to exhibit superior performance of no phase segregation, no color aging, improved stability, good reproducibility, and simple device fabrication procedure, etc. Therefore, the exploration of new SMWLEs is of great importance and attractive. SMWLEs are normally achieved by mixing either two complementary colors (blue and yellow) or three primary colors (red, green, and blue). According to the origin of these mixed color bands, they can be divided into three classes including (1) pure fluorescent SMWLEs whose emission originates only from singlet excitons, generated in such as monomer/excimer complex^7, 8^, excited-state intramolecular proton transfer systems^9, 10^, prompt/delayed dual fluorescence^11, 12^, and conformation-dependent emission systems^13^, etc., (2) hybrid fluorescent/phosphorescent SMWLEs stem from radiative decay of both singlet and triplet excitons^14^, and (3) pure phosphorescent SMWLEs that emit only from triplet excitons^3, 15, 16^. Although dye molecules with phosphorescence can utilize three quarters of the electrically generated excitons for light emission and they are thus promising energy-efficient lighting sources^17^, to date there are limited metal-free examples of hybrid SMWLE^14^ and pure-phosphorescent SMWLE^16^.

The search for pure organic phosphorescent SMWLE remains at the early stage and a challenging research area because pure organic (metal-free) phosphors are scarce and triplet excitons are easily quenched. As well known, metal-free phosphors are rare and generally exhibit dim phosphorescence under ambient conditions because of inefficient spin-orbit coupling, the long-lived sensitive triplet excitons and quenching by impurity with long lifetimes. Recently, materials with room temperature phosphorescence (RTP)^18, 19^ have redrawn extensive interest because of their potential applications in optics^20^, electronics^21^, and biological area^22^. On one hand, to obtain efficient pure organic RTP, we need first to populate triplet excitons by enhancing intersystem crossing and meanwhile suppress the nonradiative dissipation. Several groups have employed different methodologies including polymer aggregation^23^, crystallization^24–31^, halogen bonding^32^, host-guest composition^33^, self-assembly^34^, H-aggregation^35^, polymer matrix assisting^36, 37^, molecule-metal hybrid^38^, and metal-organic framework hosting^39^, etc. to develop efficient pure organic RTP systems.

**Fig. 1:**
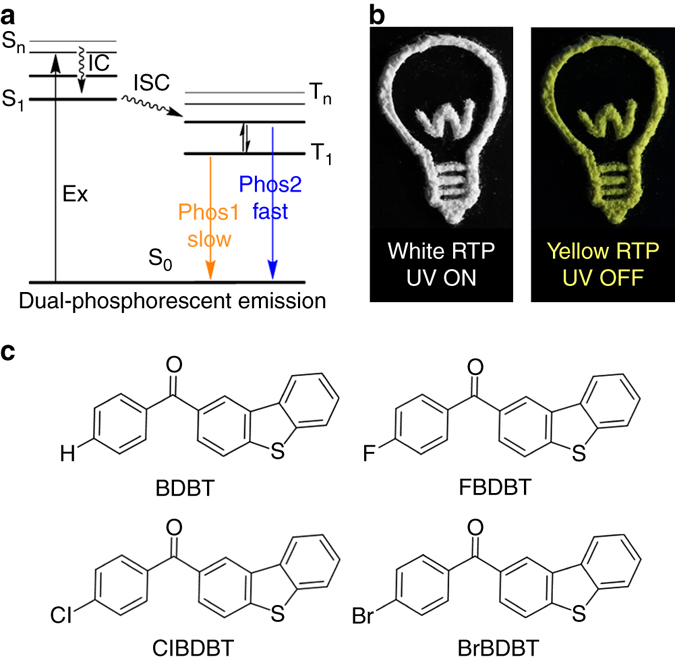
Strategy and example to develop single molecule white light-emitting RTP system. **a** Jablonski diagram for dual phosphorescent emission. **b** Photo-pattern of ClBDBT. A lamp was drawn using powder of ClBDBT. The white lamp was taken when excitation source is on, the yellow lamp was taken when excitation source is off. **c** Molecular structures of room temperature phosphors studied here

On the other hand, to achieve pure-phosphorescent SMWLE, the phosphors need to show at least two distinct phosphorescent emission bands for color mixing. In principle, if the phosphors could decay radiatively from both higher triplet state (e.g., T_2_) and the lowest triplet state (T_1_)^40–42^, the chance to observe dual phosphorescence and the possibility to obtain white light will be largely enhanced (Fig. 1a). The phosphorescence from the upper excited state occurs in two ways from the higher triplet state, i.e., T_2_, one is not involving the lower triplet state T_1_ and the other is thermally populated from T_1_. The former one requires large energy separation between those two states. The later one requires that T_1_ and T_2_ lie in energetic proximity or have small energy gap and are strong vibronically mixed or thermally equilibrated^42–45^. As early reported, the later one is the most promising T_2_ phosphorescence, in which much faster radiative decay rate of T_2_ than that of T_1_ is also needed to counterbalance the smaller proportion of T_2_^42–45^. As a result, both T_2_ and T_1_ undergo radiative decay to generate dual phosphorescence. Because of these tough requirements, so far no pure phosphorescent SMWLE has been achieved based on pure organic molecules.

Here we report four pure organic phosphors containing carbonyl group (C=O), heavy halogen atom (F, Cl, or Br), and π-extended dibenzothiophene subunit as shown in Fig. 1c. They all show dual RTP emission in the crystalline state. Interestingly, pure phosphorescent white emission is observed in ClBDBT as shown in Fig. 1b with Commission Internationale de l’Éclairage 1931 coordinates of (0.33, 0.35). The white light emission comes from the mixing of two RTP bands with different wavelengths and lifetimes, which originate from two electronic excited triplet states with different excitation energies and transition orbital features. We also demonstrate that the intrasystem mixing of dual RTP arising from low-lying and high-lying triplet states emission is a versatile strategy to achieve metal-free pure phosphorescent SMWLEs.

## Results

### Synthesis

Benzophenone is an archetypal phosphor and exhibits fast emission (~ms) both in solution at 77 K^46^ and in crystalline state at room temperature^24, 47^. On the contrary, dibenzothiophene (DBT) is a persistent (~s) phosphor at 77 K^48^ and exhibits weak dual fluorescence/phosphorescence emission in the crystal state at room temperature (Supplementary Fig. 1). Through incorporation of benzoyl chlorides into dibenzothiophene by the Friedel-Crafts acylation, here four arylphenone derivatives, namely, BDBT, FBDBT, ClBDBT and BrBDBT (Fig. 1c), were afforded. Note that both the lone pair electrons in carbonyl group and the heavy halogen atom can trigger efficient intersystem crossing to boost the inherent phosphorescence efficiency. The π-extended dibenzothiophene subunit will introduce varied triplet exited states with different molecular orbital configurations and energy levels and leads dual phosphorescence decay^49^. These molecules are found to be non-luminescent in solutions (Supplementary Fig. 2), but emit intensely as crystals at ambient conditions. This suggests that crystallization has induced emission^24^, which is a common phenomenon observed in aggregation-induced emission luminogens^50^. As purity is crucial for photophysical properties, we first purify them by column chromatography and then two-time recrystallization from chloroform-hexane solution is used. Last, the elemental analysis, melting points and high performance liquid chromatography are applied to check their purity before photophysical property measurement (Supplementary Fig. 3).

### Photophysical property

**Fig. 2:**
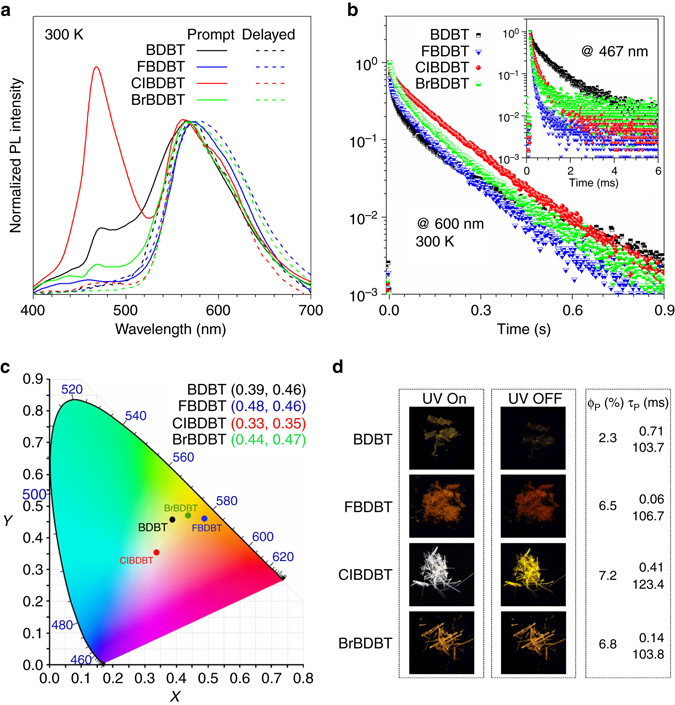
Photophysical properties of BDBT, FBDBT, ClBDBT and BrBDBT. **a** The prompt (*solid line*) and delayed (*dash line*, 10 ms) PL spectra of the crystalline powders of BDBT, FBDBT, ClBDBT, and BrBDBT at 300 K. **b** PL decay curves of **1**–**4** measured at 600 nm for persistent emission and at 467 nm for fast emission (*inset*) at 300 K. **c** CIE 1931 coordinates of prompt emission of BDBT, FBDBT, ClBDBT, and BrBDBT. **d** Photographs of BDBT, FBDBT, ClBDBT, and BrBDBT taken before and after the removal of excitation source and their phosphorescence quantum yields and lifetimes. The excitation was 365 nm

Figure 2a shows the steady-state (prompt) photoluminescence (PL) spectra (solid lines) of the crystalline powders of BDBT, FBDBT, ClBDBT, and BrBDBT. These compounds are found to exhibit dual emission bands peaked at around 470 and 570 nm. Since the PL spectra (wavelength) of BDBT, FBDBT, ClBDBT, and BrBDBT and their UV-vis absorption spectra (Supplementary Fig. 4) are similar, the halogen atoms exert little effect on the molecular energy levels. The *dashed lines* in Fig. 2a describe the time-resolved (delayed) PL spectra obtained 10 ms after excitation. Under this condition, the prompt and short-lived emission are unlikely to be detected. The delayed spectra of these molecules show almost a single band at ~570 nm, revealing the emission bands at ~470 nm are short-lived emission. Therefore, the short-lived and long-lived species are both involved in their light emission process. The emission at longer wavelength region have longer lifetime.

To confirm this, the time-resolved decay curves at 467 and 600 nm were recorded (Fig. 2b). The emission at longer wavelength region shows lifetimes in the second scale. On the contrary, PL at 467 nm decays in a much faster fashion of milisecond (Fig. 2b, *inset* and Supplementary Fig. 5). The slow emission components show lifetimes of ~0.1 s and are undoubtedly associated with phosphorescence. The fast emission components have lifetimes ranging from 0.06 to 0.71 ms, but they are also phosphorescence in nature because the nanosecond decay properties of the molecules exhibit no any signals (Supplementary Fig. 6). They are neither prompt fluorescence nor delayed fluorescence. They should originate from the radiative decay from the higher triplet state in agreement with its higher energy. Such claim will be further verified vide infra.

The Commission Internationale de l’Éclair-age (CIE) 1931 color space is the well-known defined quantitative links between physical pure colors and physiological perceived colors in human color vision^51^. The pure white color has the CIE chromaticity coordinates of (0.33, 0.33). The CIE chromaticity coordinates of BDBT, FBDBT, ClBDBT and BrBDBT calculated from their steady-state PL spectra are plotted in Fig. 2c, which cover the emission color of yellow, orange, and white. Note that ClBDBT shows the CIE coordinates of (0.33, 0.35), which are quite close to the value of pure white color. Indeed, as shown in Figs. 1b and 2d, crystals of ClBDBT exhibit bright white appearance upon UV irradiation with an overall phosphorescence quantum yield (Φ_P_) of 7.2%. After the removal of the excitation source, the crystals show yellow emission, whose intensity fades slowly with time, which is in consistent with the persistence nature of the PL at longer wavelength. The Supplementary Movie 1 shows the appearance of the emitting crystals of ClBDBT when UV light is on and off. Meanwhile, BDBT crystals emit yellow light with a Φ_P_ of 2.3%, the crystalline powders of FBDBT show a more intense orange light with a higher Φ_P_ of 6.5%. On the other hand, BrBDBT crystals emit yellow light with a Φ_P_ of 6.8%, suffering no appearance change in the absence of the excitation source, indicating its dominant-persistent phosphorescence feature. Their detailed photophysical properties were summarized in Supplementary Table 1.

**Fig. 3:**
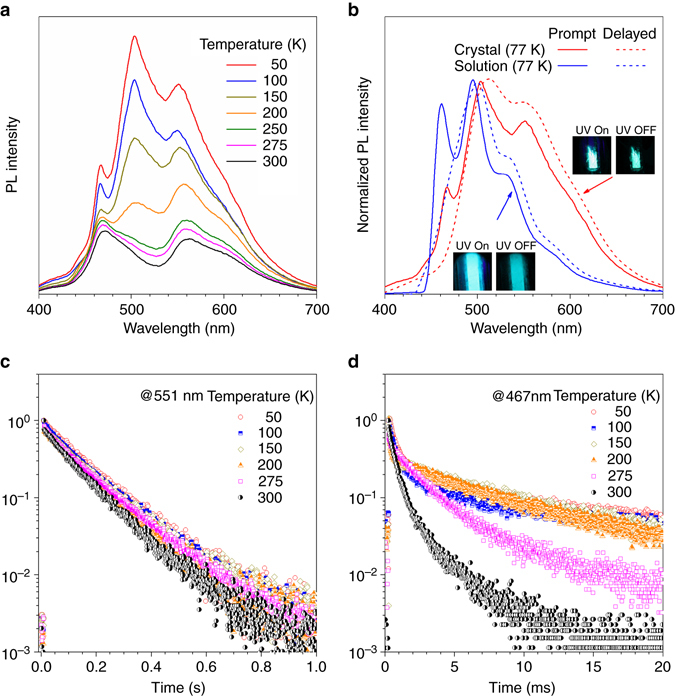
Temperature-dependent photophyscial properties of ClBDBT. **a** PL spectra of ClBDBT crystals measured at different temperatures from 50 to 300 K. **b** The prompt (*solid line*) and delayed (*dash line*, 10 ms) PL spectra and photos of ClBDBT crystals and solution (1 mM) in THF at 77 K. *Inset*: Photos of crystal and solution of ClBDBT taken at 77 K before and after removal of the UV excitation source. **c**, **d** Time-resolved PL decay curves of ClBDBT measured at **c** 551 nm and **d** 467 nm from 50 to 300 K

To verify the origin of the dual emission, we investigated temperature effect on the PL of ClBDBT. With a decrease in the temperature from 300 to 50 K, the PL at 467 nm and at 551 nm increase by one- and three-fold, respectively. The increase of overall PL intensity attributes to the suppression of nonradiative decay of the excited states and the portion of the lowest triplet state is expected to increase due to the thermal equilibrium at low temperature. Meanwhile, a new sharp peak emerges at 503 nm less than 250 K (Fig. 3a). It is assigned as the 0-0 peak of T_1_ phosphorescence in the following theoretical calculations, which is always smeared out owing to the thermal broadening at high temperature^52^.

The steady-state PL spectra (solid line) of crystal, amorphous powder, and solution measured at 77 K show similar spectral profiles, all having three emission bands with different relative intensity and the longest emission peaks (Fig. 3b). The difference should originate from their varied morphologies of solution, amorphous, and crystalline state. The intermolecular interactions and crystal packing modes influence the emission wavelength and intensity. The situations are identical to those of BDBT, FBDBT, and BrBDBT (Supplementary Figs. 8–9). Therefore, the study still focuses on their molecular structure-property relationship investigation. The disappearance of the emission band at 467 nm in the delayed PL spectra (dash line) reveals its short-lived feature and is well correlated with the lifetime result shown in Fig. 2b. The almost identical photographs of crystal and solution of ClBDBT shown in the *inset* of Fig. 3b suggest that the emission at 467 nm contributes only a small proportion to the overall PL at low temperature, decreasing from 34% at 300 K to 13% at 50 K (Supplementary Fig. 10).

The emission bands at 503 and 551 nm belong to the same excited states for their similar persistent lifetimes (Fig. 3c and Supplementary Fig. 11). The PL decay curves measured at 551 nm at different temperature are similar, demonstrating temperature-independent persistent lifetime (Fig. 3c). In contrast, those measured at 467 nm are sensitive to temperature (Fig. 3d). The lifetime is increased with decreasing the temperature, which rules out the assignment of the emission band at 467 nm to thermally activated delayed fluorescence (TADF)^53^, but assumed as T_2_ phosphorescence. At ambient temperature, the upper-lying T_2_ state is populated by thermal equilibrium with the T_1_ state. Its fast decay rate then give rise to phosphorescence with short lifetime. Although nonradiative decay is suppressed at low temperature, the proportion of T_2_ state becomes smaller at lower temperature. Thus, only a small increase in the PL intensity at 467 nm and a small contribution to the overall PL were observed upon temperature drop.

### Theoretical calculation

**Fig. 4:**
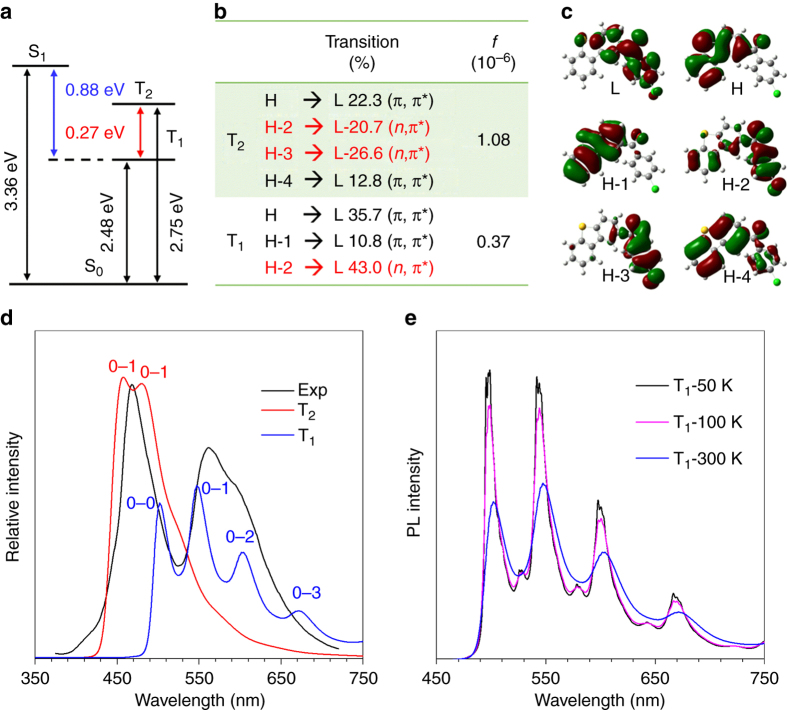
Theoretical calculations for mechanistic investigation. Calculated **a** adiabatic energy levels, **b** electronic transition characters, and **c** involved frontier molecular orbitals, and emission spectra of **d** T_1_ and T_2_ states at 300 K and **e** T_1_ state at different temperature from 50 to 300 K of ClBDBT at (TD) B3LYP/6-31(d)/GAFF level. Experimental spectrum (Exp.) is given for comparison

To gain deeper insights into the mechanism of the dual phosphorescence, theoretical calculations were carried out for the studied compounds in crystalline phase. The aggregation effect was considered by using the hybrid quantum mechanics and molecular mechanics (QM/MM) approach. The computation models were built by digging a big supercell with 5 × 5 × 5 unit cells from the X-ray crystal structures^54^. The computational details can be found in Supplementary Information. The calculated energy levels, electronic transition characters, and molecular orbitals (H for highest occupied molecular orbital and L for lowest unoccupied molecular orbital) for the lowest two triplet states (T_1_ and T_2_) in crystals of ClBDBT (Chart S1), BDBT, and BrBDBT are summarized in Fig. 4a and Supplementary Figs. 14 and 15.

Taking ClBDBT for example, it is easily seen from Fig. 4a, c that (1) there are two lowest excited triplet states (T_1_ and T_2_) below the lowest excited single state (S_1_), which indicate T_1_ and T_2_ both have chances to be bestowed the excited state energy from S_1_. The adiabatic energy difference between T_2_ and T_1_ is 0.27 eV, which is larger than the experimental value of 0.19 eV. On the other hand, the large energy difference (0.88 eV) between S_1_ and T_1_ suggests that TADF is less likely to occur at room temperature^55^; (2) both T_1_ and T_2_ states are the mixed electronic states with (*n*,π*) and (π,π*) transitions owing to the contributions from lone pairs of oxygen and conjugated electrons among planar DBT or phenyl groups; (3) while the T_2_ state is dominated by (*n*,π*) transition as revealed by its large transition coefficients, the T_1_ state possesses a more (π,π*) transition, which would result in larger spin-orbit coupling between T_2_ and S_0_ states than that between T_1_ and S_0_ states according to the El-Sayed rule^56^; (4) the (π,π*) transition exhibits apparent charge transfer feature from π-conjugated dibenzothiophene (H and H-1) unit to benzophenone moiety (L), whereas the (*n*,π*) transition always localizes on the benzophenone unit involving the participation of deeper orbitals (H-2 and H-3), which lead to the higher-energy T_2_ with (*n*,π*) transition character and lower-energy T_1_ with (π,π*) transition character. As known, the direct electronic dipole transition from a pure triplet to singlet state is forbidden and it becomes allowed when considering the spin-orbit coupling between triplet and singlet states. As a result, the transition oscillator strength (f) of T_2_ is larger by one order of magnitude than that of T_1_, which leads to short-time lifetime of T_2_ and long-time lifetime of T_1_ (Fig. 4b).

In order to confirm the origin of the dual phosphorescence, we further calculated the vibronically resolved emission spectra of T_1_ and T_2_ states, respectively, by using the thermal vibration correlation function spectrum theory in MOMAP program^57–59^ (see Fig. 4d). Comparing the calculated and experimental emission spectra at 300 K in Fig. 4d, both the position and profile are well matched, which indicates the adopted computational method and theory in this work are suitable to describe the photophysical property of the studied systems. The emission spectra of T_1_ and T_2_ behave as different fine structures. The T_1_ emission spectrum displays four peaks, which is assigned as 0-0 peak, and 0-1, 0-2, and 0-3 of high-frequency (1500–1750 cm^−1^) C-C or C=O stretching vibration modes (Supplementary Fig. 17). While the T_2_ emission spectrum demonstrates two comparable peaks in intensity, which are ascribed to the 0-1 of low-frequency (90–130 cm^−1^) out-of-plane vibration modes and 0-1 of high-frequency normal modes. The 0-0 peak is much weaker than the 0-1 peaks in intensity and smeared out owing to the strong vibronic coupling and temperature effect (Supplementary Fig. 17). Based on these results, the emission bands in experimental PL spectra are classified to evaluate the energy levels of electronic states. To better understand the temperature dependence of the spectral fine structure, we plotted the emission spectra arising from T_1_ state at different temperatures. With temperature increasing, the spectrum is broadening and the intensity is decreasing gradually. At low temperature, the 0–0 peak is dominant. While at high temperature, the vibration satellites overwhelm the 0–0 peak and become main peaks. Strikingly, the temperature dependence of spectral shape is well reflected by the experimental spectrum locating at 503-560 nm from 300 to 50 K, indicating that it originates from the decay of T_1_ state and the peak at 503 nm belong to 0–0 vibration character^52^.

### Crystal structure

**Fig. 5:**
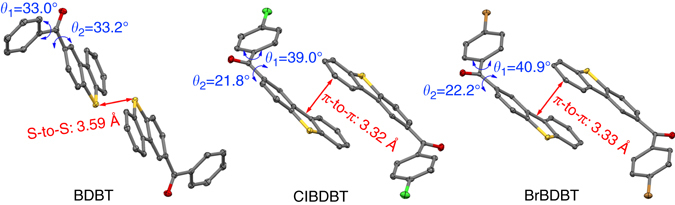
Crystal structures of BDBT, ClBDBT and BrBDBT. (Carbon, oxygen, sulfur, chlorine, and bromine atoms are shown as *gray*, *red*, *yellow*, *green*, and *orange* ellipsoids at the 50% probability level, and hydrogen atoms are removed for clarity)

Single crystals of BDBT, ClBDBT, and BrBDBT qualified for X-ray crystallography are grown from slowly evaporation of chlorofrom-hexane solutions. BDBT is plate and ClBDBT and BrBDBT are needles (Fig. 2d). Attempts to grow the single crystals of FBDBT for X-ray crystallography analysis was tried but failed as the crystalline plates are too thin. The details of their crystal structures are given in Supplementary Table 2. The crystalline phase of BDBT is orthorhombic with space group *Pbca*. The crystal structures of ClBDBT and BrBDBT adopt monoclinic with space group *P*2_1_ */n* and the lattice dimensions are almost the same between the two compounds. All of them exist in the dimeric units linked by S−S interaction in BDBT, π−π interactions in ClBDBT and BrBDBT with almost the same π−to−π distance. The interactions within the dimeric units should help the intermolecular electronical coupling of excited-state configurations in the crystals and their photophysical properties^29^. For example, the π−π interactions redshift the emission peaks in crystals compared to their solutions (Fig. 3b and Supplementary Figs. 8 and 9). The dihedral angles between the aryl groups and carbonyl subunits are also depicted in Fig. 5. ClBDBT have the smallest dihedral angle (21.8°) between the DBT and C=O, suggesting the best conjugation in the molecule. Therefore, the (*n*,π*) from the C=O and (π,π*) could exhibit good communication and mix with each other to form the hybrid excited-states triplet states.

### Stability

As well known, aryl ketones are subjects that can easily degrade on exposure to UV irradiation in solution. The aging of the molecule may lead color drifting or change. Fortunately, the white emission of ClBDBT is obtained at crystalline state. The dense packing of molecule within the crystal lattice will not only block the quenching of oxygen and moisture, but also prevent the degradation of molecules from inner part under ambient conditions^60^. To test the stability, crystalline powder of ClBDBT was exposed to UV irradiation for 12 h under air at room temperature. The HPLC measurement reveals that the sample is robust and no degrade byproduct (i.e., DBT) is found (Supplementary Fig. 18). The PL spectra also suggests that the sample is stable enough toward multiple PL scan (Supplementary Fig. 19).

As shown in Supplementary Fig. 20, the intensity of the peaks at 467 and 551 nm becomes lower when they are transformed into partial crystalline samples or amorphous powders by external mechanical grinding or melting followed by cooling at ambient conditions, the overall white emission of pristine crystals of ClBDBT is still persistent. The response behaviors toward external stimulus is different from dual emission, which consist of fluorescence or delayed fluorescence^11, 12, 14^. This due to the fact that both forms emit phosphorescence from T_1_ and T_2_ states, which show similar response to the external perturbations.

## Discussion

After confirming the origin of emission band, we realize mixing dual RTP arising from the low- and high-lying triplet states should be a general strategy to achieve metal-free pure phosphorescent SMWLE. As the two states have the same multiplicity, we call the strategy Intrasystem Mixing. This is the first SMWLE example applying such strategy. The success attributes to proper molecular structure engineering that integration of the archetypal phosphor benzophenone with dibenzothiophene. The junction mode is a decisive point to make the T_1_ and T_2_ have the required molecular orbital configurations. Here a phenyl group is shared between benzophenone and dibenzothiophene motif. Benzophenone mainly provides 3(*n*,π)* orbital which results fast emission, and dibenzothiophene mainly provides 3(π,π)* orbital which facilitates the slow radiative decay. The proper mixing results in the molecular orbitals getting a hybrid contribution from both components.

Normally, phosphors emit from the lowest triplet state T_1_ according to the Kasha’s rule. Only limited examples of molecules show phosphorescence from higher triplet states. Furthermore, most of the observed T_2_ phosphorescence is considered to originate from the thermal population from the T_1_ state. Therefore, the Δ*E*(T_1_−T_2_) value is normally quite small to make T_2_ have enough proportion at room temperature. According to the Boltzmann distribution, T_2_ still have smaller proportion than T_1_ but the room temperature can still provide the essential activation energy. Then, a much faster radiative decay rate of T_2_ is required to reverse the situation and make T_2_ and T_1_ have the balanced emission intensity. Based on El-Sayed’s rule, T_2_ must have mainly 3(*n*,π)* orbital character.

Following our strategy, we successfully found another SMWLE example, which is also a halogenated benzophenone (4-bromo-4′-chlorobenzophenone, BCBP). BCBP emits cool white light upon UV irradiation with CIE coordinates of (0.26, 0.30) and a high quantum yield of 28.6% (Supplementary Table 1). The decay curves measured at 425 and 550 nm reveal two emission bands that have two quite different lifetimes of 0.19 and 19.2 ms, respectively (Supplementary Fig. 12). After temperature-dependent photophysical studies and theoretical calculations (Supplementary Fig. 16), the similar results (time-resolved decay profiles and calculated triplet orbitals) prove that it is an another Intrasystem Mixing SMWLE that arises from dual RTP emissions of the T_1_ and T_2_ (Supplementary Fig. 13). So, this is a good proof-of-concept example of the proposed strategy.

Because it is quite difficult to obtain two emission bands accurately in a proper ratio to result white emission, here we reported these two cases of pure organic phosphorescent SMWLE by utilizing T_2_ emission. The effect of halogen atoms in tuning the ratio of phosphorescence from T_1_ and T_2_ is critically important to obtain the white light. From the examples discussed above, it is quite difficult to figure out as they exert little effect on the molecular energy levels and packing modes. Possibly, they influence the intramolecular conformation and intermolecular electronic coupling and the effect is subtle but comprehensive. Also, the packing modes and intermolecular interactions in solid states play subtle and complex effects on the photophysical behaviors of these dyes. Efforts to decipher these effects are still in progress.

In summary, five pure organic room temperature phosphors with dual RTP emission are reported. Experimental and theoretical investigations reveal that the dual RTP should arise from the low- and high-lying triplet states. Based on this, a design strategy for achieving white light emission materials with pure phosphorescent was demonstrated. The design strategy gained from our experimental and theoretical discussions will allow for the exploration of white emission organic phosphors. A single molecule white light emitter (ClBDBF) was explored with CIE 1931 coordinates of (0.33, 0.35). The efforts to explore more SMWLEs followed this strategy and to decipher the effects of halogen atoms and packing modes are in progress.

### Data availability

The data that support the findings of this study are available from the authors on reasonable request, see author contributions for specific data sets. The X-ray crystallographic coordinates for the structures reported in this article have been deposited at the Cambridge Crystallographic Data Centre (CCDC) under deposition numbers CCD 1556843, 1556844, and 1556845. These data can be obtained free of charge from The Cambridge Crystallographic Data Centre via www.ccdc.cam.ac.uk/data_request/cif.

## Electronic supplementary material

Supplementary Information

Supplementary Movie 1

## References

[1] Kamtekar KT, Monkman AP, Bryce MR (2010). Recent advances in white organic light-emitting materials and devices (WOLEDs). Adv. Mater..

[2] Farinola GM, Ragni R (2011). Electroluminescent materials for white organic light emitting diodes. Chem. Soc. Rev..

[3] Ni W-X (2013). Approaching white-light emission from a phosphorescent trinuclear Gold(I) cluster by modulating its aggregation behavior. Angew. Chem. Int. Ed..

[4] Han M, Tian Y, Yuan Z, Zhu L, Ma B (2014). A phosphorescent molecular “butterfly” that undergoes a photoinduced structural change allowing temperature sensing and white emission. Angew. Chem. Int. Ed..

[5] Higuchi T, Nakanotani H, Adachi C (2015). High-efficiency white organic light-emitting diodes based on a blue thermally activated delayed fluorescent emitter combined with green and red fluorescent emitters. Adv. Mater..

[6] Du M (2016). Novel emitting system based on a multifunctional bipolar phosphor: an effective approach for highly efficient warm-white light-emitting devices with high color-rendering index at high luminance. Adv. Mater..

[7] Yang Q-Y, Lehn J-M (2014). Bright white-light emission from a single organic compound in the solid state. Angew. Chem. Int. Ed..

[8] Chen Y-H (2016). Insight into the mechanism and outcoupling enhancement of excimer-associated white light generation. Chem. Sci..

[9] Yang Y (2006). An organic white light-emitting fluorophore. J. Am. Chem. Soc..

[10] Tang K-C (2011). Fine tuning the energetics of excited-state intramolecular proton transfer (ESIPT): White light generation in a single ESIPT system. J. Am. Chem. Soc..

[11] Xie Z (2015). White-light emission strategy of a single organic compound with aggregation-induced emission and delayed fluorescence properties. Angew. Chem. Int. Ed..

[12] Xu B (2016). Achieving remarkable mechanochromism and white-light emission with thermally activated delayed fluorescence through the molecular heredity principle. Chem. Sci.

[13] Zhang Z (2015). Excited-state conformational/electronic responses of saddle-shaped N,N′-disubstituted-dihydrodibenzo[a,c]phenazines: wide-tuning emission from red to deep blue and white light combination. J. Am. Chem. Soc..

[14] Mao Z (2015). Linearly tunable emission colors obtained from a fluorescent–phosphorescent dual-emission compound by mechanical stimuli. Angew. Chem. Int. Ed..

[15] Wong KM-C, Yam VW-W (2011). Self-assembly of luminescent alkynylplatinum(II) terpyridyl complexes: modulation of photophysical properties through aggregation behavior. Acc. Chem. Res..

[16] Shao S, Ding J, Wang L, Jing X, Wang F (2012). White electroluminescence from all-phosphorescent single polymers on a fluorinated poly(arylene ether phosphine oxide) backbone simultaneously grafted with blue and yellow phosphors. J. Am. Chem. Soc..

[17] Han C (2012). Controllably tuning excited-state energy in ternary hosts for ultralow-voltage-driven blue electrophosphorescence. Angew. Chem. Int. Ed..

[18] Vo-Dinh, T. *Room Temperature Phosphorimetry for Chemical Analysis* (John Wiley, 1984).

[19] Hurtubise, R. J. *Phosphorimetry: Theory, Instrumentation and Applications* (VCH, 1990).

[20] Hirata S, Totani K, Yamashita T, Adachi C, Vacha M (2014). Large reverse saturable absorption under weak continuous incoherent light. Nat. Mater..

[21] Sun H (2014). Smart responsive phosphorescent materials for data recording and security protection. Nat. Commun..

[22] Zhang G, Palmer GM, Dewhirst MW, Fraser CL (2009). A dual-emissive-materials design concept enables tumour hypoxia imaging. Nat. Mater..

[23] Zhang G (2007). Multi-emissive difluoroboron dibenzoylmethane polylactide exhibiting intense fluorescence and oxygen-sensitive room-temperature phosphorescence. J. Am. Chem. Soc..

[24] Yuan WZ (2010). Crystallization-induced phosphorescence of pure organic luminogens at room temperature. J. Phys. Chem. C.

[25] Bergamini G (2013). A persulfurated benzene molecule exhibits outstanding phosphorescence in rigid environments: from computational study to organic nanocrystals and OLED applications. J. Mater. Chem. C.

[26] Fermi A (2014). Molecular asterisks with a persulfurated benzene core are among the strongest organic phosphorescent emitters in the solid state. Dyes Pigm.

[27] Li C (2015). Reversible luminescence switching of an organic solid: controllable on–off persistent room temperature phosphorescence and stimulated multiple fluorescence conversion. Adv. Opt. Mater..

[28] Gong Y (2015). Achieving persistent room temperature phosphorescence and remarkable mechanochromism from pure organic luminogens. Adv. Mater..

[29] Yang Z (2016). Intermolecular electronic coupling of organic units for efficient persistent room-temperature phosphorescence. Angew. Chem. Int. Ed..

[30] Shimizu M, Kimura A, Sakaguchi H (2016). Room-temperature phosphorescence of crystalline 1,4-Bis(aroyl)-2,5-dibromobenzenes. Eur. J. Org. Chem..

[31] Shimizu M (2016). Siloxy group-induced highly efficient room temperature phosphorescence with long lifetime. J. Phys. Chem. C.

[32] Bolton O, Lee K, Kim HJ, Lin KY, Kim J (2011). Activating efficient phosphorescence from purely organic materials by crystal design. Nat. Chem.

[33] Hirata S (2013). Efficient persistent room temperature phosphorescence in organic amorphous materials under ambient conditions. Adv. Funct. Mater..

[34] Fermi A, Bergamini G, Roy M, Gingras M, Ceroni P (2014). Turn-on phosphorescence by metal coordination to a multivalent terpyridine ligand: a new paradigm for luminescent sensors. J. Am. Chem. Soc..

[35] An Z (2015). Stabilizing triplet excited states for ultralong organic phosphorescence. Nat. Mater..

[36] Kwon MS (2015). Suppressing molecular motions for enhanced room-temperature phosphorescence of metal-free organic materials. Nat. Commun..

[37] Chen X (2016). Versatile room-temperature-phosphorescent materials prepared from N-substituted naphthalimides: emission enhancement and chemical conjugation. Angew. Chem. Int. Ed..

[38] Yang X, Yan D (2016). Strongly enhanced long-lived persistent room temperature phosphorescence based on the formation of metal–organic hybrids. Adv. Opt. Mater..

[39] Mieno H, Kabe R, Notsuka N, Allendorf MD, Adachi C (2016). Long-lived room-temperature phosphorescence of coronene in zeolitic imidazolate framework ZIF-8. Adv. Opt. Mater..

[40] Chu S-Y, Goodman L (1975). A simple theoretical model for dual phosphorescence. Chem. Phys. Lett..

[41] Prieto MFR, Nickel B, Grellmann KH, Mordziński A (1988). Dual phosphorescence from 2-(2′-hydroxyphenyl)benzoxazole due to keto-enol tautomerism in the metastable triplet state. Chem. Phys. Lett..

[42] Chaudhuri D (2013). Metal-free OLED triplet emitters by side-stepping Kasha’s rule. Angew. Chem. Int. Ed..

[43] Wagner PJ, May MJ, Haug A, Graber DR (1970). Phosphorescence of phenyl alkyl ketones. J. Am. Chem. Soc..

[44] Wagner PJ, Kemppainen AE, Schott HN (1973). Effects of ring substituents on the type II photoreactions of phenyl ketones. How interactions between nearby excited triplets affect chemical reactivity. J. Am. Chem. Soc..

[45] Itoh T (2004). Successive occurrence of the T1(π, π*) and T2(n, π*) phosphorescence and the S1(n, π*) fluorescence observed for p-cyanobenzaldehyde in a solid matrix. J. Lumin..

[46] Kearns DR, Case WA (1966). Investigation of singlet→triplet transitions by the phosphorescence excitation method. III. Aromatic ketones and aldehydes. J. Am. Chem. Soc..

[47] Kuzmanich G (2011). Excited state kinetics in crystalline solids: self-quenching in nanocrystals of 4,4′-disubstituted benzophenone triplets occurs by a reductive quenching mechanism. J. Am. Chem. Soc..

[48] Siegel S, Judeikis HS (1966). Triplet state zero-field splittings of some structurally related aromatic hydrocarbon and heterocyclic molecules1. J. Phy. Chem..

[49] Zhao W (2016). Rational molecular design for achieving persistent and efficient pure organic room-temperature phosphorescence. Chem.

[50] Mei J, Leung NLC, Kwok RTK, Lam JWY, Tang BZ (2015). Aggregation-induced emission: together we shine, united we soar!. Chem. Rev..

[51] Smith T, Guild J (1931). The C.I.E. colorimetric standards and their use. Trans. Opt. Soc..

[52] Becker, R. S. *Theory and Interpretation of Fluorescence and Phosphorecence*, 218 (Wiley Interscience, 1969).

[53] Lee SY, Yasuda T, Yang YS, Zhang Q, Adachi C (2014). Luminous butterflies: efficient exciton harvesting by benzophenone derivatives for full-color delayed fluorescence OLEDs. Angew. Chem. Int. Ed..

[54] Ma H (2016). Electrostatic interaction-induced room-temperature phosphorescence in pure organic molecules from QM/MM calculations. J. Phys. Chem. Lett..

[55] Leitl MJ, Krylova VA, Djurovich PI, Thompson ME, Yersin H (2014). Phosphorescence versus thermally activated delayed fluorescence. Controlling singlet–triplet splitting in brightly emitting and sublimable Cu(I) compounds. J. Am. Chem. Soc..

[56] El-Sayed MA (1968). Triplet state. Its radiative and nonradiative properties. Acc. Chem. Res..

[57] Shuai, Z. G., Peng, Q., Niu, Y. L. & Geng, H. *MOMAP, A Free and Open-Source Molecular Materials Property Prediction Package*, Revision 0.2.004. Available at http://www.shuaigroup.net/ (Shuai Group, 2014).

[58] Niu Y, Peng Q, Deng C, Gao X, Shuai Z (2010). Theory of excited state decays and optical spectra: application to polyatomic molecules. J. Phys. Chem. A.

[59] Peng Q, Niu Y, Shi Q, Gao X, Shuai Z (2013). Correlation function formalism for triplet excited state decay: combined spin–orbit and nonadiabatic couplings. J. Chem. Theory Comput..

[60] Simoncelli S, Kuzmanich G, Gard MN, Garcia-Garibay MA (2010). Photochemical reaction mechanisms and kinetics with molecular nanocrystals: surface quenching of triplet benzophenone nanocrystals. J. Phys. Org. Chem..

