# Supplementary material for: White light emission from a single organic molecule with dual phosphorescence at room temperature
Source: Nat Commun. 2017 Sep 4;8:416. doi: 10.1038/s41467-017-00362-5 (PMC5583377; doi:10.1038/s41467-017-00362-5)
Supplement: Supplementary file 1 — Supplementary Information [file 41467_2017_362_MOESM1_ESM.pdf]

Description of Supplementary Files

File Name: Supplementary Information
Description: Supplementary Methods, Supplementary Discussion, Supplementary Figures, Supplementary Tables and Supplementary References

File Name: Supplementary Movie 1
Description: The appearance of the emitting crystals of ClBDBT when UV light is on and off.

Supplementary Methods

All the chemicals and reagents were purchased from Aldrich and used as received without further purification. All the molecules synthesized were purified by column chromatography and recrystallization from dichloromethane and hexane for two times, and fully characterized by ^{1}H NMR, ^{13}C NMR and high resolution mass spectroscopies and elementary analysis.
^{1}H and ^{13}C NMR spectra were recorded on a Bruker AV 400 Spectrometer at 400 and 100 MHz in $CDCl_3$, respectively. Tetramethylsilane was used as the internal standard. High-resolution mass spectra (HRMS) were recorded on a GCT premier CAB048 mass spectrometer operating in MALDI-TOF mode. Elementary analysis was performed on a Thermo Finnigan Flash EA1112. Gel filtration chromatography was performed using a ZORBAX SB-C18 column (Agilent) conjugated to an Agilent 1260 Infinite HPLC system. Before running, each sample was purified via 0.22 μm filter to remove any aggregates. The flow rate was fixed at 1.0 mL/min, the injection volume was 20 μL and each sample was run for 6 min. The absorption wavelength used was set at 330 nm. 100 % percent of acetonitrile was used as the running buffer. The photoluminescence spectra were measured on a PerkinElmer LS 55 spectrophotometer. The lifetime, time-resolved excitation spectra, steady state and time-resolved emission spectra, temperature dependent photoluminescence spectra and absolute luminescence quantum yield were measured on a Edinburgh FLSP 920 fluorescence spectrophotometer equipped with a xenon arc lamp (Xe900), a microsecond flash-lamp (uF900), a picosecond pulsed diode laser (EPL-375), a closed cycle cryostate (CS202*I-DMX-1SS, Advanced Research Systems) and an integrating sphere (0.1 nm step size, 0.3 second integration time, 5 repeats), respectively. Mean decay times (τ_P) were obtained from individual lifetimes τ_i and amplitudes a_i of multi-exponential evaluation. Powder X-Ray diffraction patterns were performed on an X'Pert PRO MPD diffractometer with Cu Kα radiation (λ = 1.5418 Å) at 25 °C (scan range: 4.5−50°). Single crystal data was collected on a Bruker Smart APEXII CCD diffractometer using graphite monochromated Cu Kα radiation (λ = 1.54178 Å). The photos and videos were recorded by a Cannon EOS 60D.

The amorphous solids of the phosphors were prepared by heating the samples to melt with a heating gun and quenching the melt with liquid nitrogen.

All the crystalline samples were obtained from slowly evaporative crystallization using hexane/chloroform mixture (3:1, v/v). To further check the purity of the solid samples, all the solid samples were dissolved in 100 % percent of acetonitrile and got sample solutions (50 μM), then run the HPLC.

To check the optical stability of powder samples, a pile of powder was exposed to a 365nm UV light (the power of UV tube is 8W) for 30 min to 12h, then the solid samples were dissolved in 100 % percent of acetonitrile and then sample solutions were prepared (50 μM), finally run the HPLC.

Mean decay times (τ_P) were obtained from individual lifetimes τ_i and amplitudes a_i of multi-exponential evaluation through:

$$\tau_P = \frac{\sum_i a_i \tau_i^2}{\sum_i a_i \tau_i}$$

Synthesis

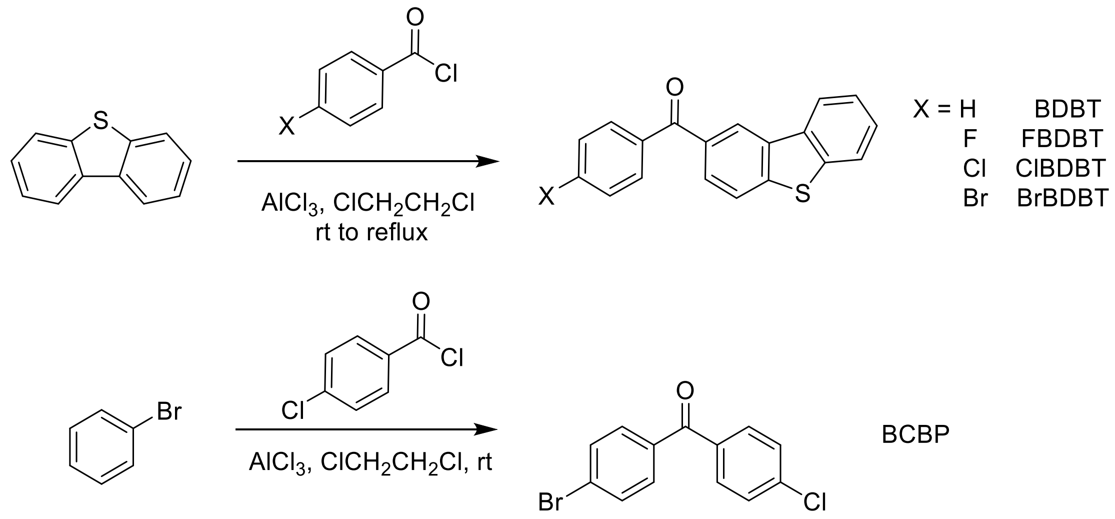

Synthetic route for BDBT, FBDBT, ClBDBT, BrBDBT and BCBP.

Dibenzo[*b*,*d*]thiophen-2-yl(phenyl)methanone (BDBT)

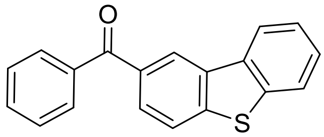

Anhydrous $AlCl_3$ (1.46 g, 11.0 mmol) in 1,2-dichloroethane (10 mL) was slowly added to benzoyl chloride (1.68 g, 12.0 mmol). The solution was stirred and kept at 0 °C. A solution of dibenzothiophene (1.84 g, 10.0 mmol) in 1,2-dichloroethane (10 mL) was then slowly injected. The reaction mixture was stirred at room temperature for 8 h and then refluxed for 2 h. The reaction was quenched with 1 N HCl in ice bath. The mixture was extracted with chloroform (3 × 100 mL). The combined organic extracts were washed with H_2O, dried over anhydrous $MgSO_4$, filtered and concentrated in vacuum. The residue was purified on a silica gel column using hexane/dichloromethane mixture (5:1, v/v) as eluent to afford the product as white powder. Yield: 87%. Melting point: 154.8-155.2℃. ^{1}H NMR (400 MHz, $CDCl_3$): δ 8.62 (d, J = 1.2 Hz, 1H), 8.20 (m, 1H), 7.90 (m, 5H), 7.65 (m, 1H), 7.52 (m, 4H). ^{13}C NMR (100 MHz, $CDCl_3$): δ 196.5, 144.1, 139.7, 138.0, 135.5, 135.2, 134.0, 132.4, 128.2, 127.4, 124.9, 123.7, 123.0, 122.6, 122.0. HRMS (MALDI-TOF, *m*/*z*): calcd. for $C_{19}H_{12}OS$, 288.3640. Found, 288.3702. Elemental analysis (calcd., found for $C_{20}H_{14}O_2$): C (79.14, 79.20), H (4.19, 4.14).

Dibenzo[*b*,*d*]thiophen-2-yl(4-fluorophenyl)methanone (FBDBT)

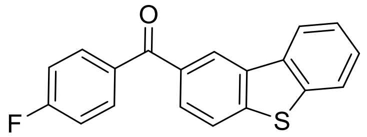

Following the same synthetic procedure for BDBT, the reaction of anhydrous $AlCl_3$ (1.46 g, 11.0 mmol), 4-fluorobenzoyl chloride (2.03 g, 10.0 mmol) and dibenzothiophene (1.84 g, 10.0 mmol) at room temperature for 12 h afforded the product as white powder. Yield: 89%. Melting point: 119.2-120.7 ℃. ^{1}H NMR (400 MHz, $CDCl_3$): δ: 8.58 (d, J = 0.8 Hz, 1H), 8.21

(m, 1H), 7.97–7.87 (m, 5H), 7.55 (m, 2H), 7.23 (t, J = 8.4 Hz, 2H). ^{13}C NMR (100 MHz, $CDCl_3$): δ 194.3, 166.0, 163.5, 143.5, 139.1, 134.8, 133.54, 133.51, 133.2, 132.1, 132.0, 127.3, 126.9, 124.3, 122.8, 122.3, 122.0, 121.3, 115.0, 114.8. HRMS (MALDI-TOF, *m*/*z*): calcd for $C_{19}H_{11}FOS$, 306.0515. Found, 306.0509. Elemental analysis (calcd., found for $C_{19}H_{11}FOS$): C (74.49, 74.45), H (6.20, 6.22).

Dibenzo[*b*,*d*]thiophen-2-yl (4-chlorophenyl)methanone (ClBDBT)

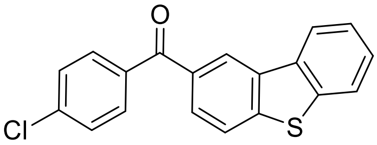

Following the same synthetic procedure for BDBT, the reaction of anhydrous $AlCl_3$ (1.46 g, 11.0 mmol), 4-chlorobenzoyl chloride (2.11g, 12.0 mmol) and dibenzothiophene (1.84 g, 10.0 mmol) at room temperature for 10 h and refluxed for 3 h afforded the product as white powder. Yield: 82%. Melting point: 138.6-139.1℃.^{1}H NMR (400 MHz, $CDCl_3$): δ: 8.58 (d, J = 1.6 Hz, 1H), 8.20 (m, 1H), 7.97–7.95 (d, 1H), 7.90–7.87 (m, 2H), 7.83–7.80 (m, 2H), 7.55–7.48 (m, 4H). ^{13}C NMR (100 MHz, $CDCl_3$): δ 195.2, 139.8, 138.9, 136.3, 135.5, 135.1, 133.6, 131.5, 128.7, 127.9, 127.5, 125.0, 123.5, 122.9, 122.7, 121.9. HRMS (MALDI-TOF, *m*/*z*): calcd for $C_{19}H_{11}ClOS$, 322.8060. Found, 322.8101. Elemental analysis (calcd., found for $C_{19}H_{11}ClOS$): C (70.70, 70.75), H (3.43, 3.31).

Dibenzo[*b*,*d*]thiophen-2-yl (4-bromophenyl))methanone (BrBDBT)

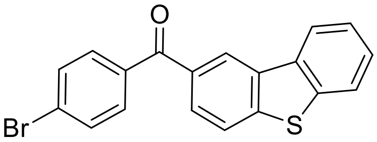

Following the same synthetic procedure for BDBT, the reaction of anhydrous $AlCl_3$ (1.46 g, 11.0 mmol), 4-bromobenzoyl chloride (2.35 g, 12.0 mmol) and dibenzothiophene (1.84 g, 10.0 mmol) at room temperature for 10 h and refluxed for 5 h afforded the product as white powder. Yield: 80%. Melting point: 135.2-135.7 ℃.

^{1}H NMR (400 MHz, $CDCl_3$): δ: 8.57 (d, J = 1.2 Hz, 1H), 8.22–8.17 (m, 1H), 7.97–7.95 (d, 1H), 7.75–7.73 (m, 2H), 7.69–7.67 (m, 2H), 7.55–7.48 (m, 4H). ^{13}C NMR (100 MHz, $CDCl_3$): δ 195.4, 144.4, 139.7, 136.7, 135.5, 135.1, 133.5, 131.6, 127.9, 127.5, 127.4, 124.9, 123.5, 123.0, 122.7, 122.0. HRMS (MALDI-TOF, *m*/*z*): calcd for $C_{19}H_{11}BrOS$, 365.9714. Found, 365.9725. Elemental analysis (calcd., found for $C_{19}H_{11}BrOS$): C (62.14, 62.20), H (3.02, 2.97).

(4-bromophenyl)(4-chlorophenyl)methanone (BCBP)

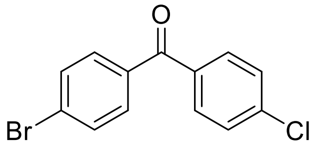

Following the same synthetic procedure for BDBT, the reaction of anhydrous $AlCl_3$ (1.46 g, 11.0 mmol), 4-chlorobenzoyl chloride (1.75 g, 10.0 mmol) and bromobenzene (2.35 g, 15.0 mmol) at room temperature for 12 h afforded the product as white powder. Yield: 95%. Melting point: 124.9-125.6 ℃.^{1}H NMR (400 MHz, $CDCl_3$): δ: 7.75 (d, J = 8 Hz, 2H), 7.66 (s, 4H), 7.49 (d, J = 8 Hz, 2H). ^{13}C NMR (100 MHz, $CDCl_3$): δ 194.4, 139.2, 135.9, 135.4, 131.8, 131.4, 131.3, 127.8. HRMS (MALDI-TOF, *m*/*z*): calcd for $C_{13}H_8BrClO$, 293.9447. Found,

293.9451. Elemental analysis (calcd., found for $C_{13}H_8BrClO$): C (52.83, 52.82), H (2.73, 2.75).

Other organic phosphorescent molecule used in this study

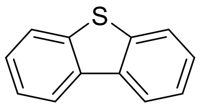

DBT

dibenzothiophene (DBT) were purchased from Sigma-Aldrich and further purified by recrystallization two times from dichloromethane and hexane.

Supplementary Discussion

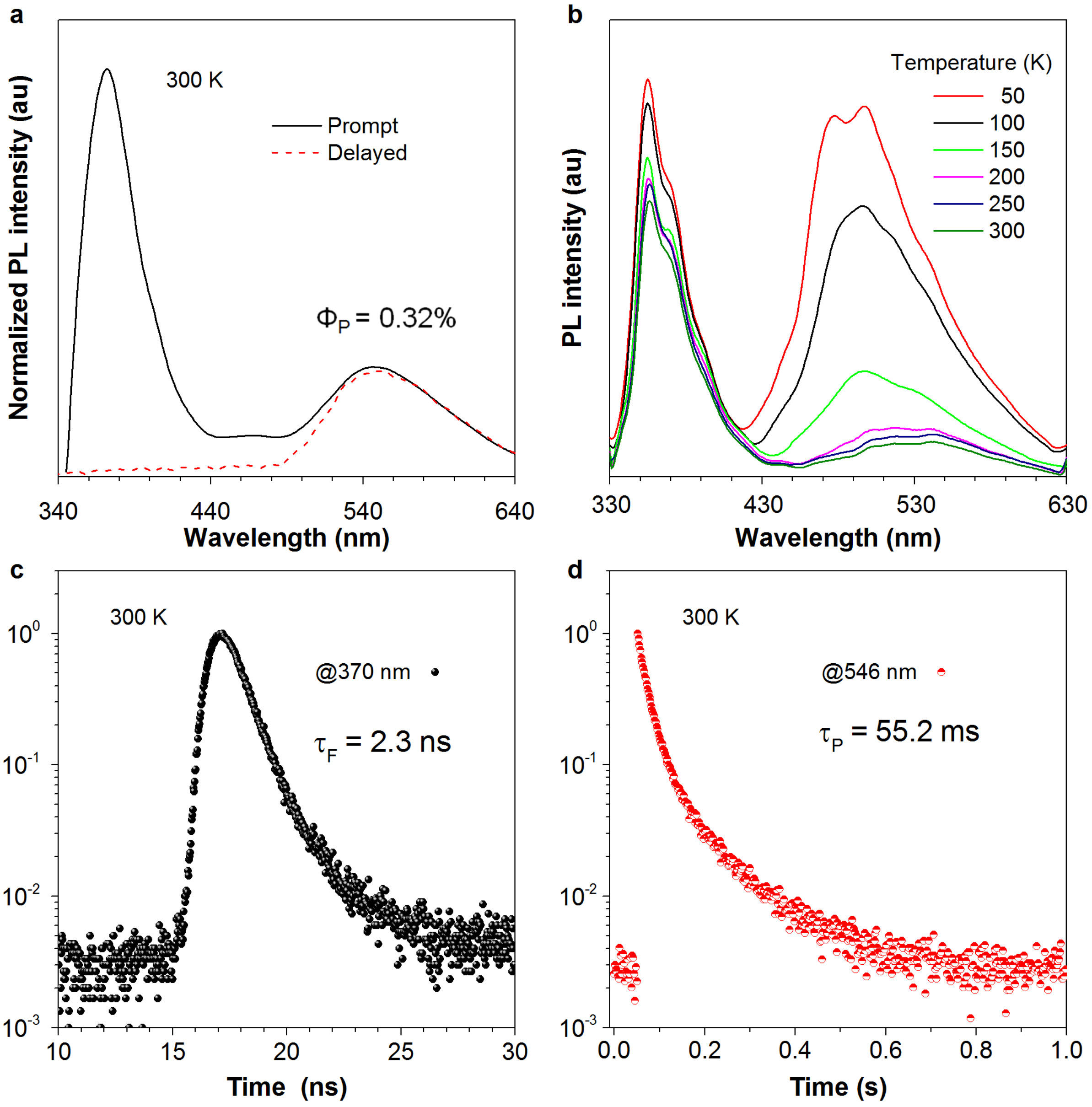

Supplementary Figure 1. (**a**), Steady-state prompt (solid) and delayed (dash, 10 ms) PL spectra of DBT. (**b**), Steady-state PL spectra measured at different temperatures (from 50 to 300 K). (**c**), Nanosecond decay profile of DBT measured at 370 nm. (**d**), Second-scale time-resolved PL decay curve measured at 546 nm. The compounds were excited at 320 nm in its crystalline state.

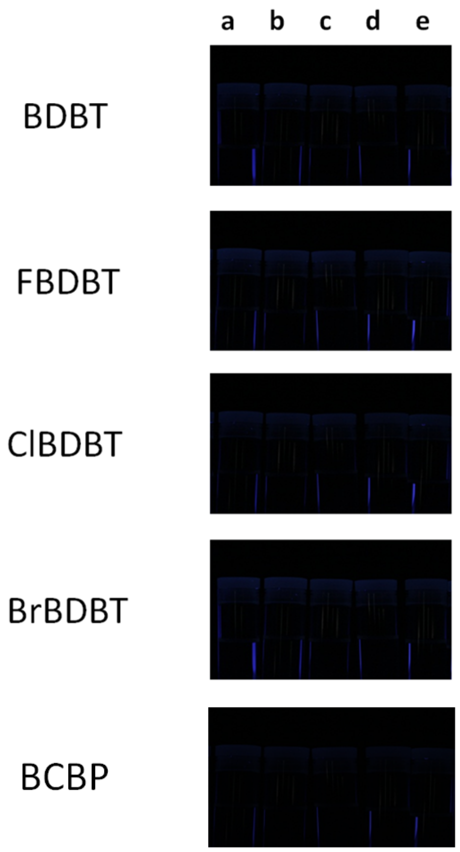

Supplementary Figure 2. Photographs of solutions in N_2-saturated (**a**) cyclohexane, (**b**) toluene, (**c**) tetrahydrofuran, (**d**) dichloromethane and (**e**) dimethylformamide taken at 300 K under UV excitation.

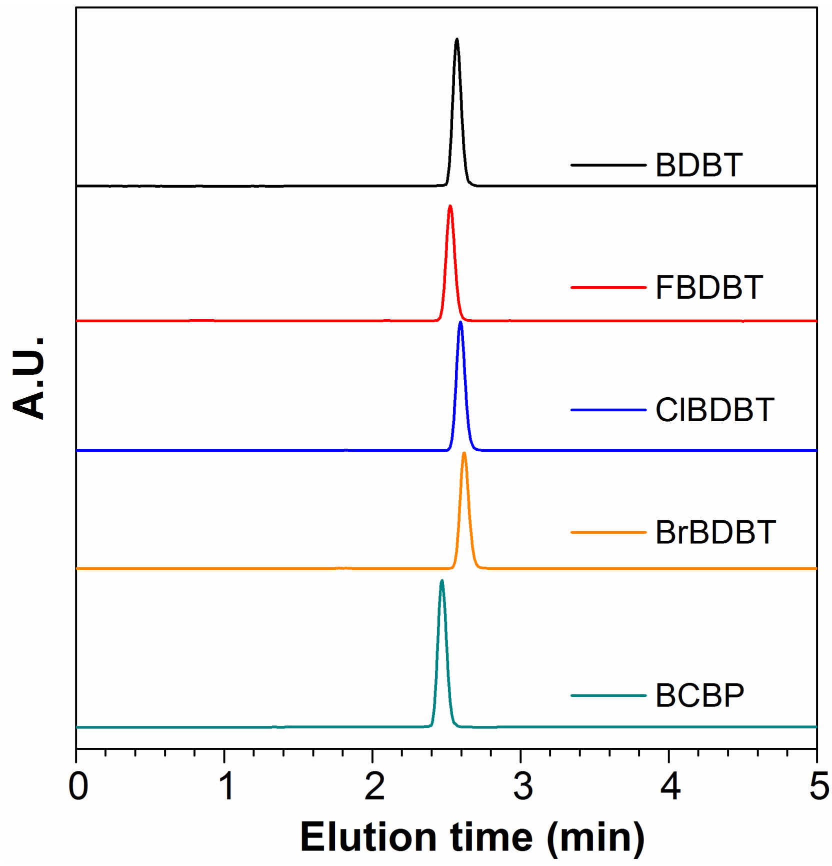

Supplementary Figure 3. High-performance liquid chromatogram spectra of BDBT, FBDBT, ClBDBT, BrBDBT and BCBP in acetonitrile solution (50 μM).

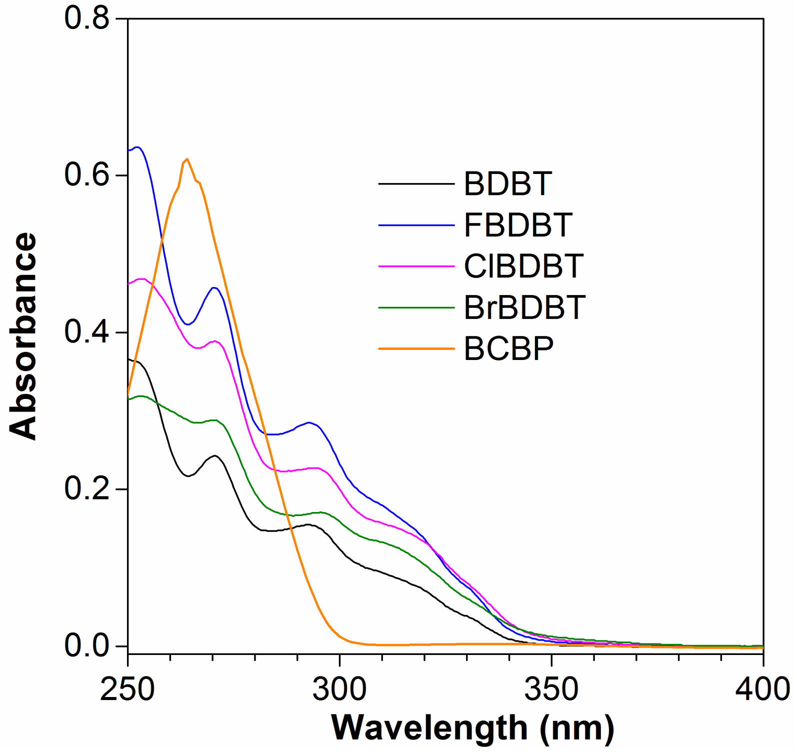

Supplementary Figure 4. UV-Vis spectra of THF solutions of BDBT, FBDBT, ClBDBT and BrBDBT. Concentration: 10^{-5} M.

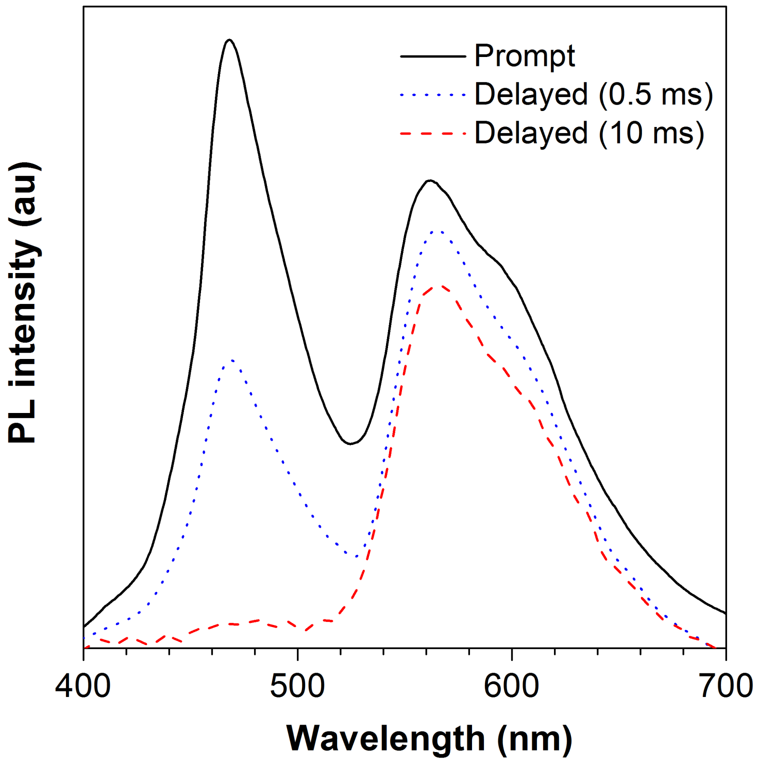

Supplementary Figure 5. Steady-state prompt (solid) and delayed (dot, 0.5 ms; dash, 10 ms) PL spectra of ClBDBT (300 K).

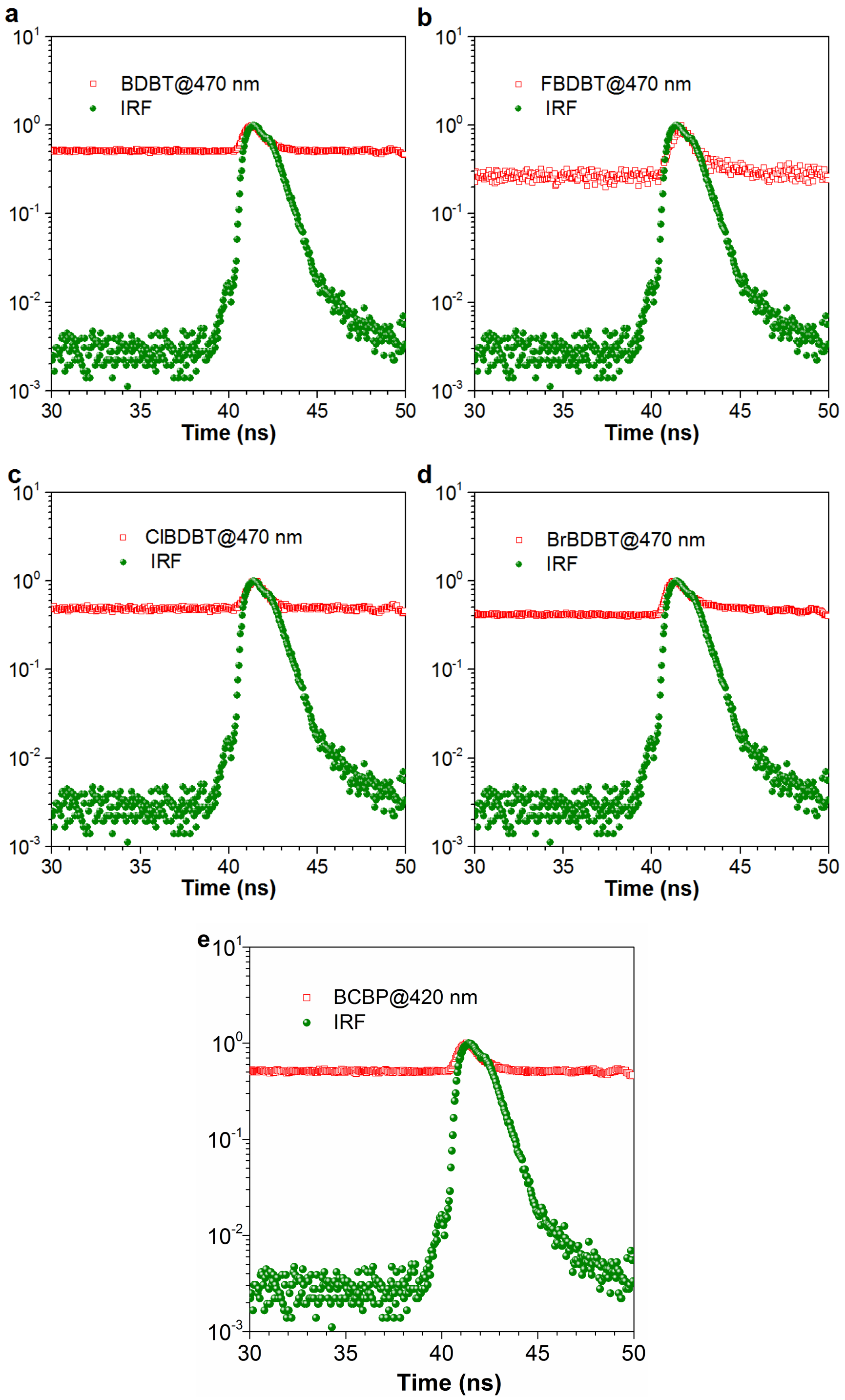

Supplementary Figure 6. Nanosecond decay profiles of BDBT (**a**), FBDBT (**b**), ClBDBT (**c**), BrBDBT (**d**) and BCBP (**e**) excited at 375 nm. A picosecond pulsed diode laser (EPL-375) was used as the light source. The impulse response function (IRF) (green) was from the output of pulsed diode laser which has a typical pulse width of less than 100 ps. All the decay profiles almost coincide with the IRF, suggesting that no obvious nanosecond fluorescence decay was detected. As persistent lifetimes were detected in all the emission bands, the photoluminescence feature of BDBT, FBDBT, ClBDBT, BrBDBT and BCBP was proved to be pure phosphorescence.

Supplementary Table 1. Photoluminescence lifetimes of DBT, BDBT, FBDBT, ClBDBT, BrBDBT and BCBP at 300 K.

compound	fluorescence	Short-lived phosphoresce			Long-lived phosphoresce		
		Wavelength (nm)	τ_i (ms)	τ_{avg} (ms)	Wavelength (nm)	τ_i (ms)	τ_{avg} (ms)
DBT	2.3 ns (370nm)		–		546	73.6(0.16) 18.6(0.84)	56.23
BDBT	–	470	0.94 (0.21) 0.10 (0.79)	0.71	567	115.5 (0.32) 10.9 (0.67)	103.7
FBDBT	–	465	0.37 (0.02) 0.05 (0.98)	0.062	565	106.7 (0.55) 11.8 (0.45)	106.7
ClBDBT	–	470	1.25 (0.02) 0.52 (0.98)	0.41	590	128.2 (0.44) 12.4 (0.56)	123.4
BrBDBT	–	467	0.70 (0.02) 0.07 (0.98)	0.14	570	113.4 (0.51) 19.2 (0.49)	103.8
BCBP	–	425	0.03 (0.94) 0.37 (0.06)	0.19	550	3.26 (74%) 25.1 (26%)	19.2

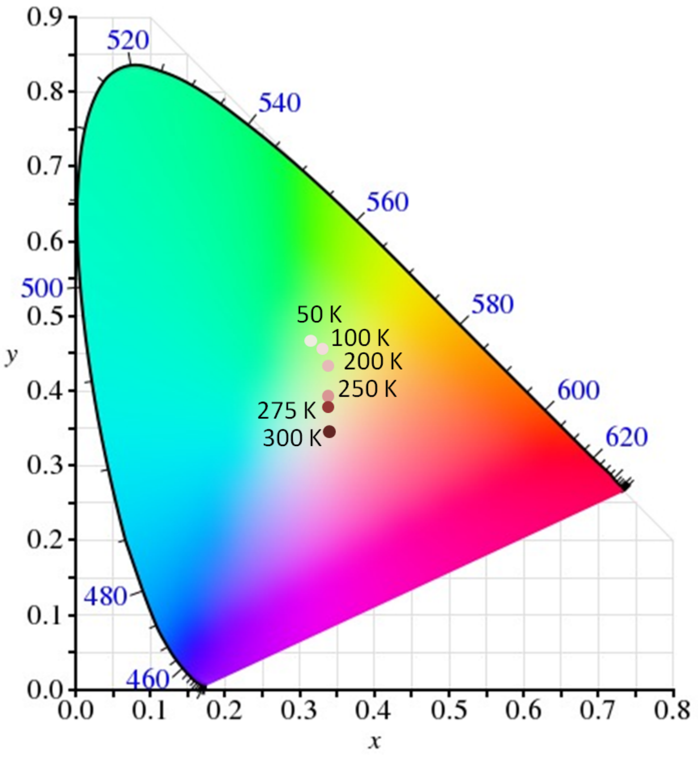

Supplementary Figure 7. CIE 1931 coordinates of the prompt emission of ClBDBT at different temperatures (from 50 to 300 K).

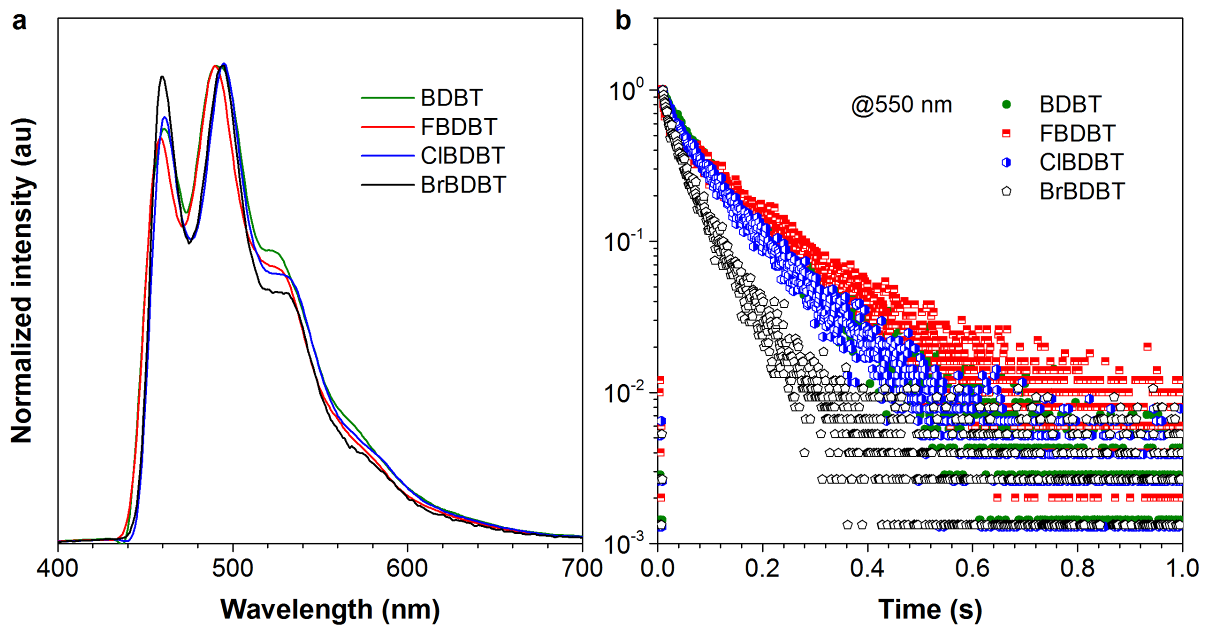

Supplementary Figure 8. (**a**), PL spectra of BDBT, FBDBT, ClBDBT and BrBDBT solution glasses measured at 77 K. (**b**), Time-resolved PL decay curves of BDBT, FBDBT, ClBDBT and BrBDBT measured at 550 nm at 77 K.

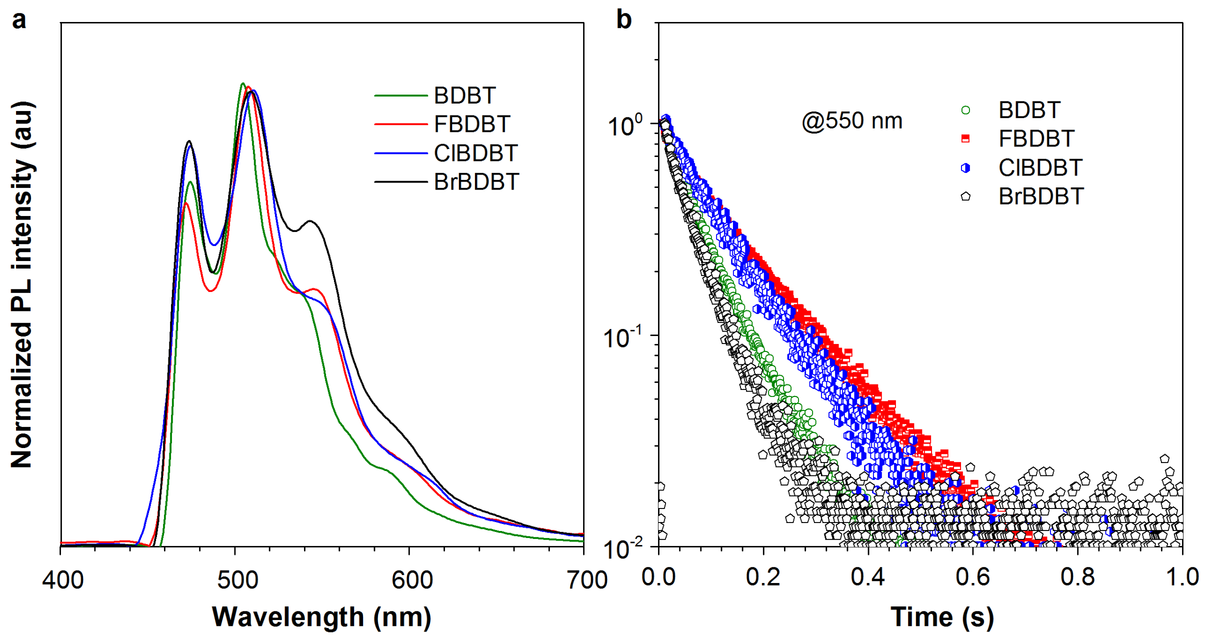

Supplementary Figure 9. (**a**), PL spectra of amorphous powders of BDBT, FBDBT, ClBDBT and BrBDBT measured at 77 K. (**b**), Time-resolved PL decay curves of BDBT, FBDBT, ClBDBT and BrBDBT measured at 550 nm at 77 K.

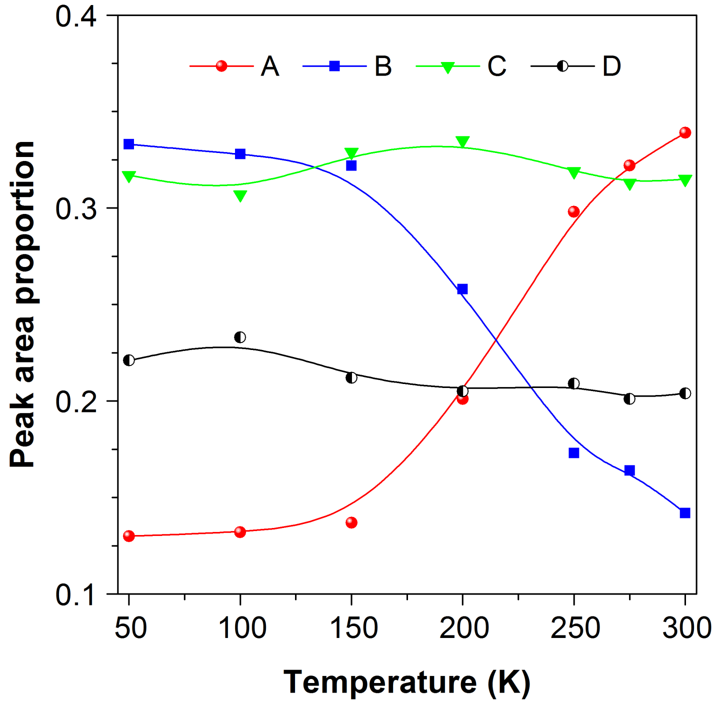

Supplementary Figure 10. Peak area proportions of the emission of ClBDBT at different temperatures (50-300 K).

Peak gravity center: A (471-474 nm), B (507-514 nm), C (553-563 nm), D (599-606 nm)

Peak area proportion: A + B + C + D = 1

Full width at half maximum: 40-43 nm

R-Square: 0.983-0.992

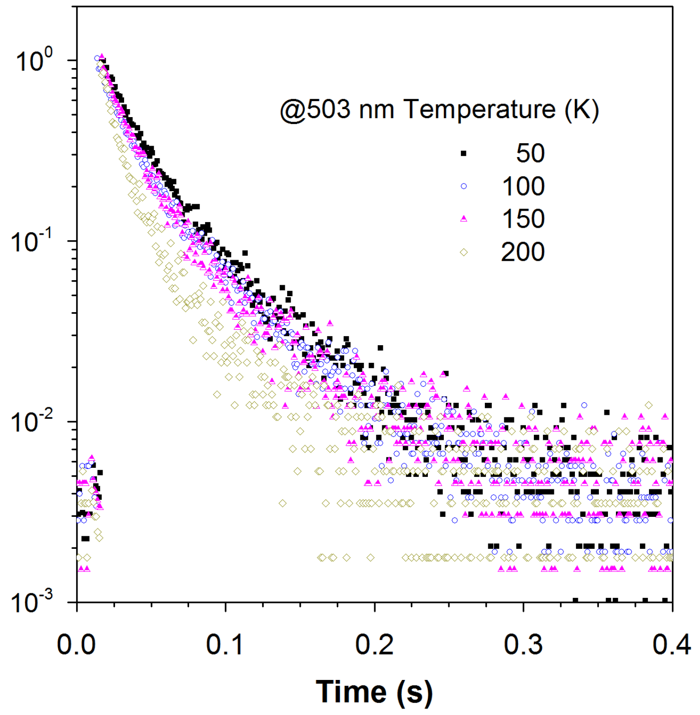

Supplementary Figure 11. PL decay curves of ClBDBT measured at 503 nm at different temperatures.

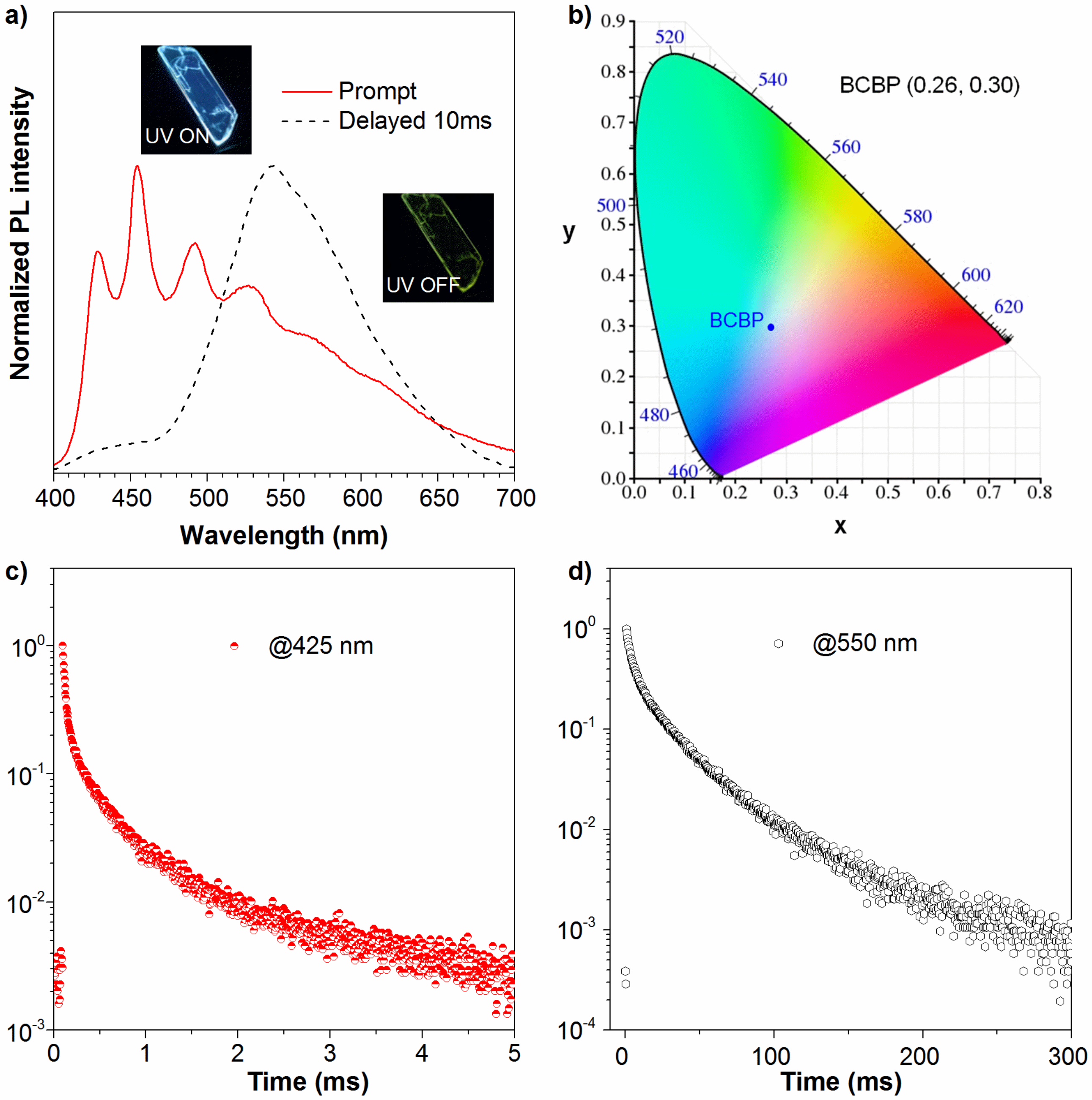

Supplementary Figure 12. (**a**),The prompt (red solid line) and delayed (black dash line, 10 ms) PL spectra of the crystalline powders of BCBP at 300 K. (**b**), CIE 1931 coordinates of prompt emission of BCBP at 300K. (**c** and **d**), PL decay curves of BCBP measured at 425 nm for fast emission (**c**) and at 550 nm for persistent emission (**d**) and at 300 K.

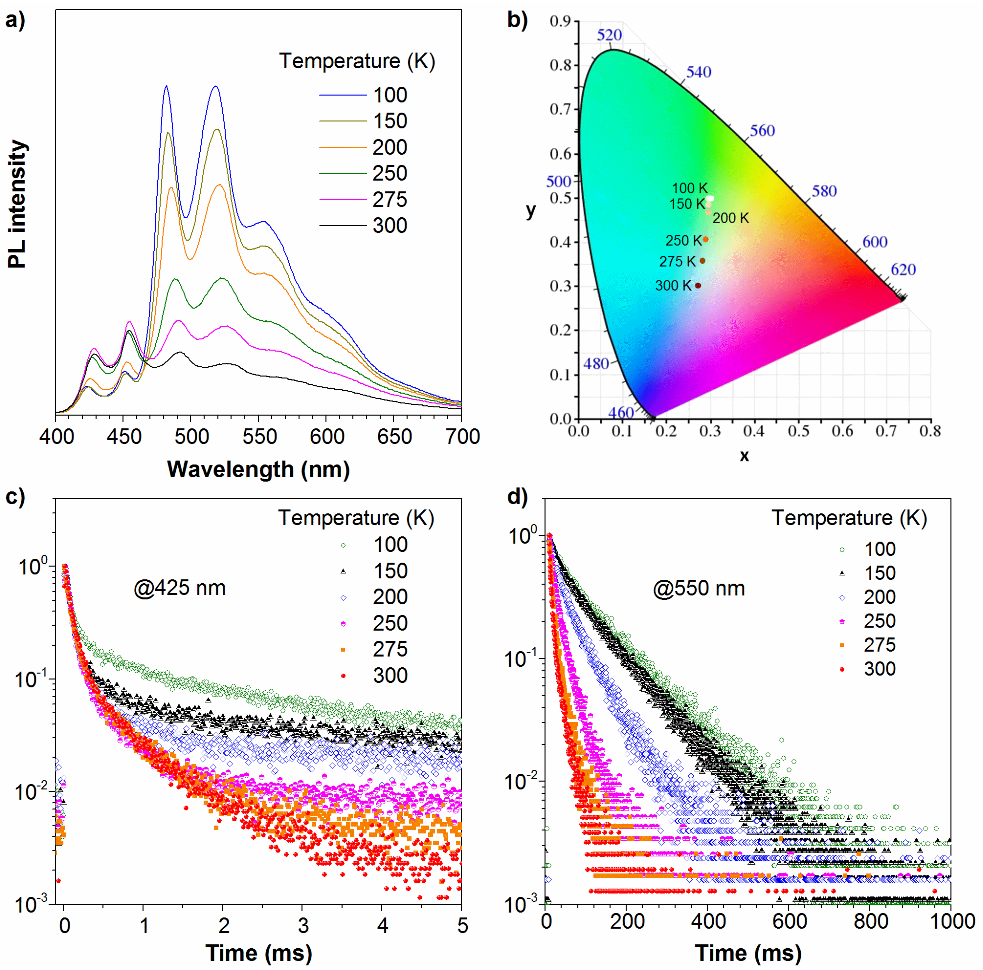

Supplementary Figure 13. (**a**), PL spectra of BCBP crystals measured at different temperatures from 100 to 300 K. (b), CIE 1931 coordinates of the prompt emission of BCBP at different temperatures (from 100 to 300 K). (**c** and **d**) Time resolved PL decay curves of BCBP measured at (**c**) 425 nm and (**d**) 550 nm from 100 to 300 K

Computational methods and results

The computational models were built from the crystal structure shown below. The quantum mechanics/molecular mechanics (QM/MM) method was implemented to deal with the electronic structures in crystal by virtue of ChemShell 3.5.[1], interfacing Turbomole 6.5[2] for QM and DL_POLY[3] with the general Amber force field (GAFF)[4] for MM. The atomic partial charges were generated by the restrained electrostatic potential (RESP)[5] method. Molecular geometry optimizations were performed for the ground state (S_0) at the level of B3LYP/6-31G(d) and for the triplet state (T_1 and T_2) at the TDDFT/B3LYP/6-31G(d) level. The excitation energies were calculated by using TDDFT for electronic excited singlet and triplet states. We further calculated the vibrational frequencies at S_0, T_1 and T_2 states at (TD)B3LYP/6-31G(d) level in order to determine the vibration emission spectra of $T_1 \rightarrow S_0$ and $T_2 \rightarrow S_0$. At the same level, the oscillator strength of triplet states is given by Beijing Density Function (BDF) program.[6-8] The emission spectra were calculated by MOMAP

program[9] with detailed formulation discribed in our previous work.[10] Cartesian coordinates were used to construct the Duschinsky matrix.

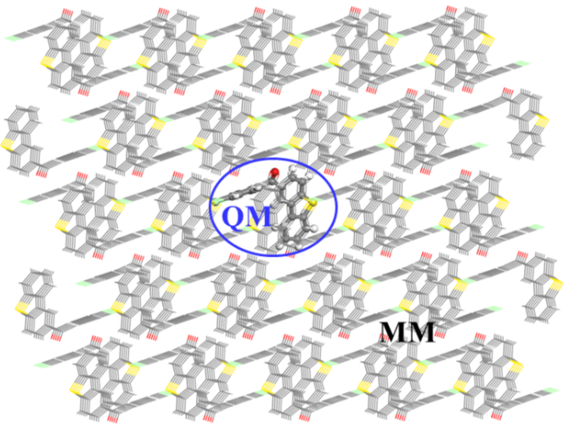

QM/MM model taking ClBDBT as an example: one central QM molecule for the higher layer and the surrounding 124 MM molecules for the lower layer.

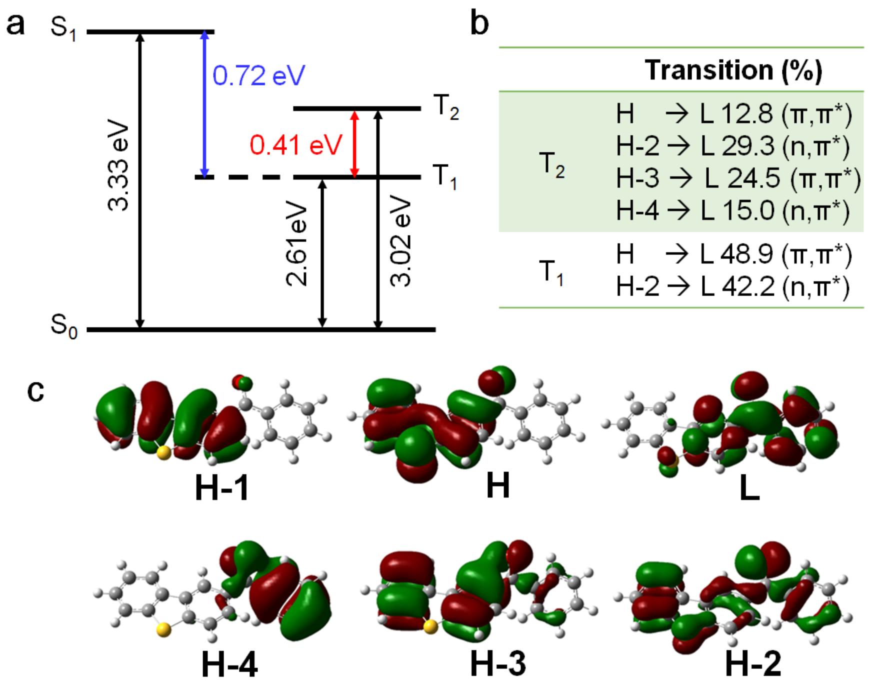

Supplementary Figure 14. Calculated adiabatic energy levels (**a**), electronic transition characters for T_1 and T_2 states (**b**) and the corresponding frontier molecular orbitals (**c**) of BDBT at B3LYP/6-31(d) level.

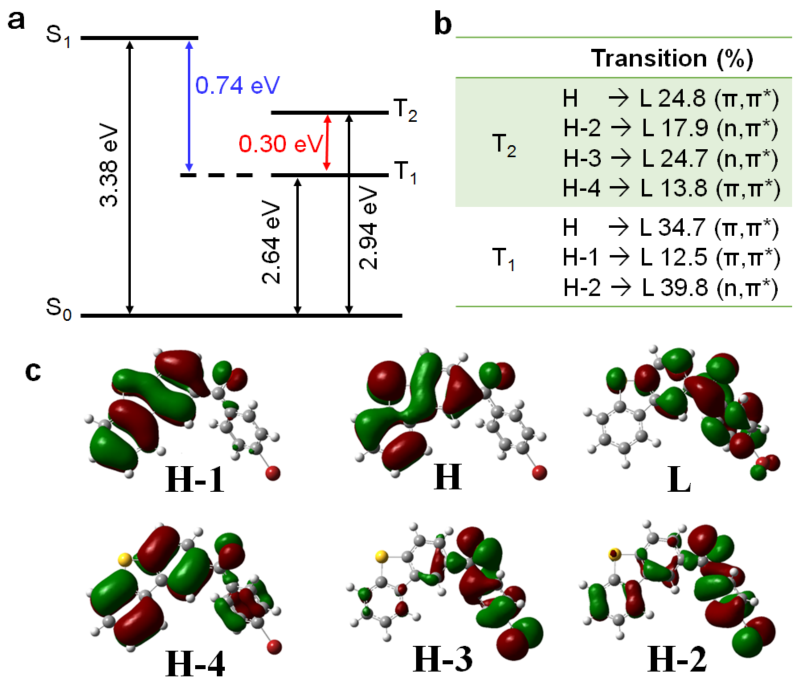

Supplementary Figure 15. Calculated adiabatic energy levels (**a**), electronic transition characters for T_1 and T_2 states (**b**) and the corresponding frontier molecular orbitals (**c**) of BrBDBT at B3LYP/6-31(d) level.

Because we failed to obtain the single crystal structure of FBDBT, no QM/MM model calculations thus could be carried out. For BDBT and BrBDBT, they have different energy gaps, electronic transition characters for T_1 and T_2 states and the corresponding frontier molecular orbitals. Briefly, BDBT has a large energy gap between T_1 and T_2 state. BrBDBT has similar energy gaps as ClBDBT, but its T_2 state show smaller (n,π*) transition.

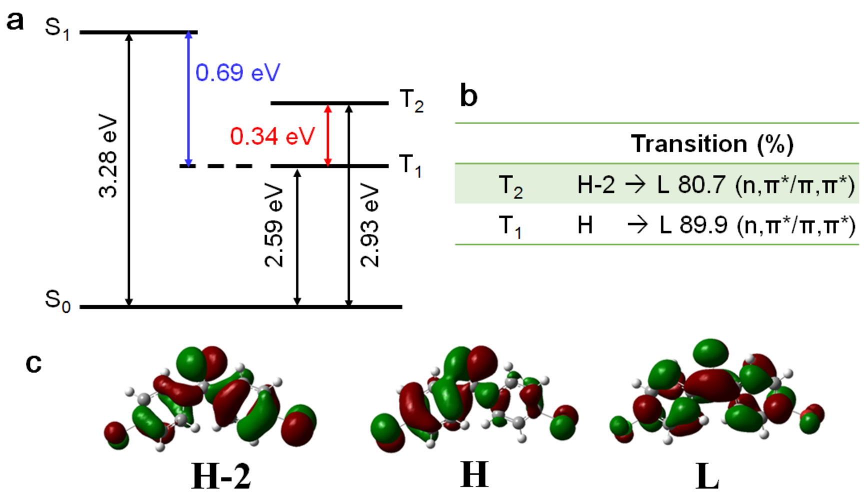

Supplementary Figure 16. Calculated adiabatic energy levels (**a**), electronic transition characters for T_1 and T_2 states (**b**) and the corresponding frontier molecular orbitals (**c**) of BCBP at B3LYP/6-31(d) level.

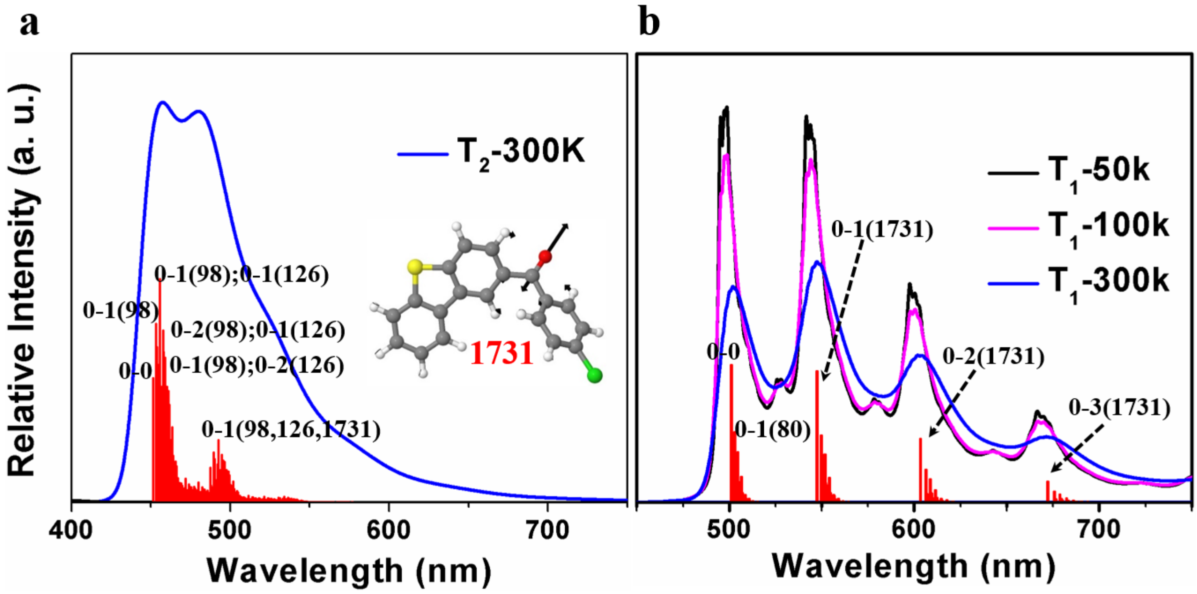

Supplementary Figure 17. Emission spectra and vibronic modes of T2 state at 300 K (**a**) and T1 state at different temperature from 50 to 300 K (**b**) of ClBDBT at (TD) B3LYP/6-31(d)/GAFF level.

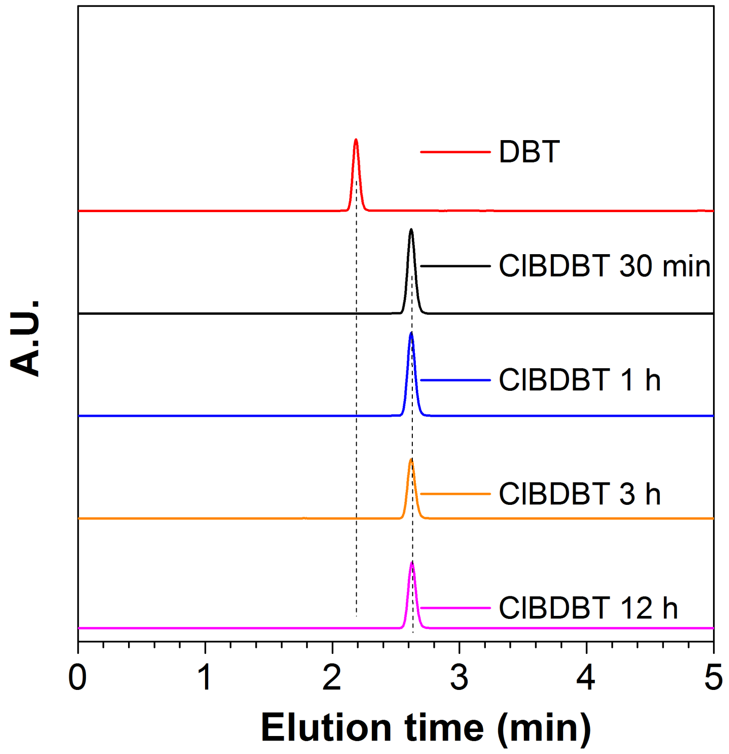

Supplementary Figure 18. High-performance liquid chromatogram spectra of DBT and ClBDBT in acetonitrile solution (50 μM). The powder of ClBDBT was exposed to UV excitation for different times (30 min - 12 h). The HPLC of ClBDBT shows that no new peak

was detected, suggesting that the powder samples is stable enough for photophysical properties measurement under air at room temperature

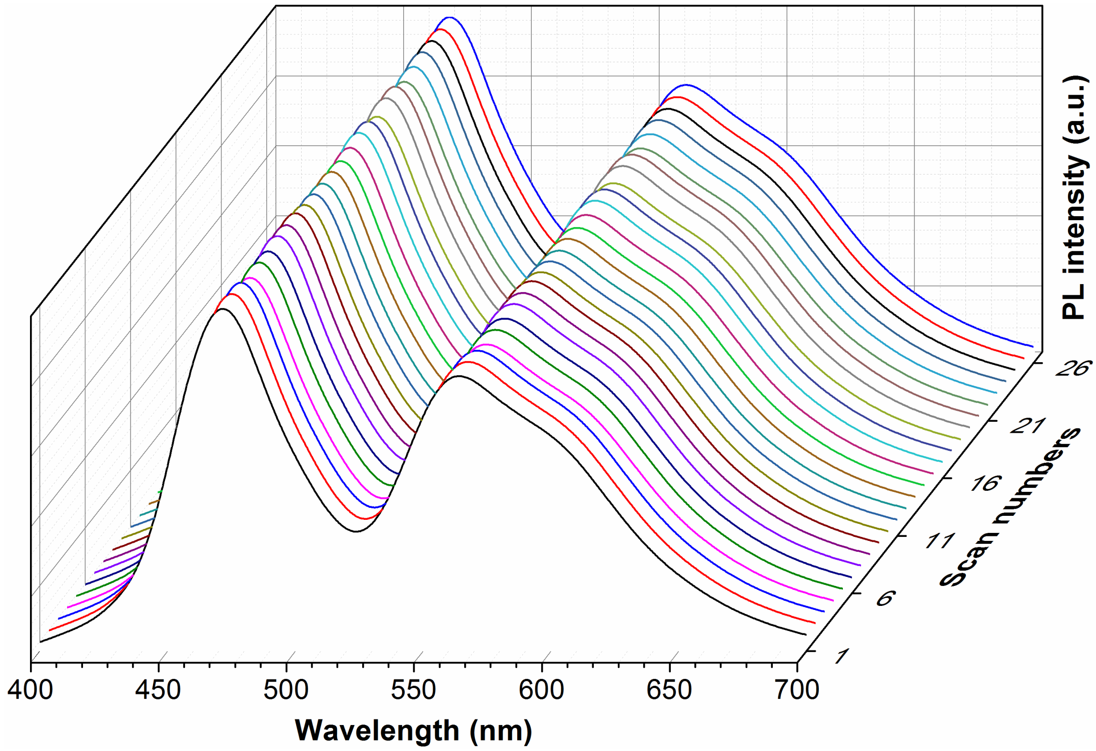

Supplementary Figure 19. PL spectra of the ClBDBT started at intervals of 5 minutes for 2 hours.

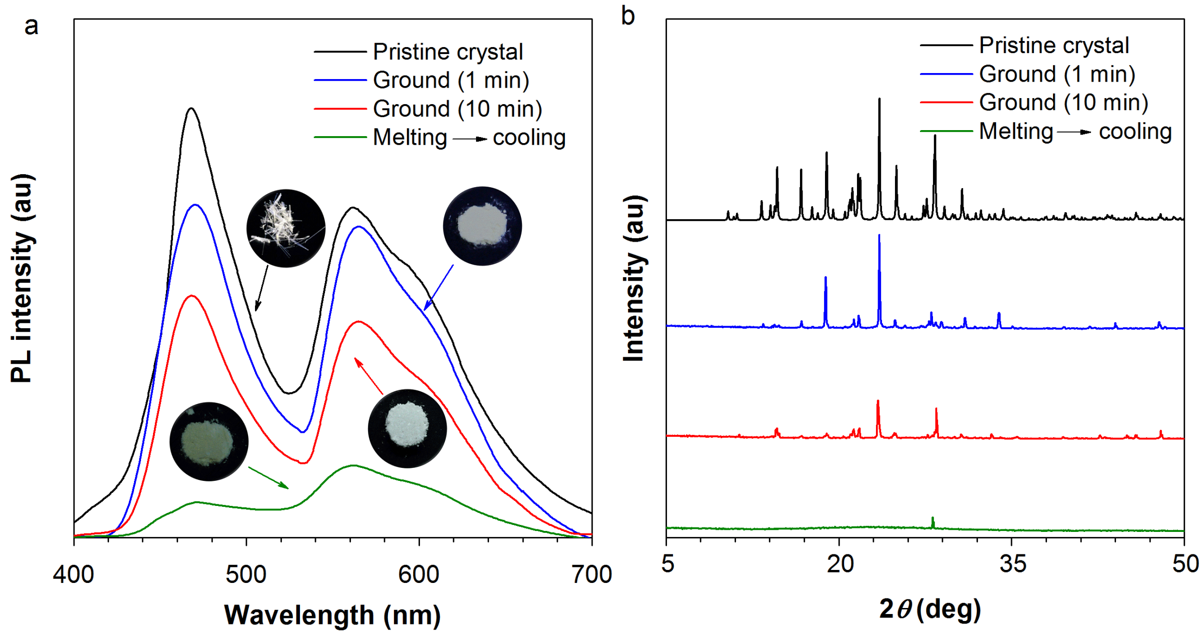

Supplementary Figure 20. (**a**), Phosphorescence spectra of ClDBT in different solid states. (**b**), Powder X-ray diffraction of ClBDBT in different solid states.

Supplementary Table 2. Crystal data and structure refinement for BDBT, ClBDBT, and BrBDBT.

Identification code	BDBT	ClBDBT	BrBDBT
Empirical formula	$C_{19}H_{12}OS$	$C_{19}H_{11}ClOS$	$C_{19}H_{11}BrOS$
Formula weight	288.35	322.79	367.25
Temperature/K	99.95(18)	100.01(10)	99.98(10)
Crystal system	orthorhombic	monoclinic	monoclinic
Space group	Pbca	$P2_1/n$	$P2_1/n$
a/Å	11.86997(13)	9.5198(2)	9.6818(3)
b/Å	8.07863(9)	9.3435(2)	9.3372(3)
c/Å	28.2144(3)	16.2983(4)	16.4006(5)
α/°	90	90	90
β/°	90	94.652(2)	95.131(3)
γ/°	90	90	90
Volume/$Å^3$	2705.57(5)	1444.93(6)	1476.68(7)
Z	8	4	4
$\rho_{calc}g/cm^3$	1.416	1.484	1.652
μ/mm^{-1}	2.067	0.406	2.923
F(000)	1200.0	664.0	736.0
Crystal size/mm^3	0.3 × 0.2 × 0.2	0.3 × 0.25 × 0.2	0.25 × 0.18 × 0.05
Radiation	CuKα (λ = 1.54184)	MoKα (λ = 0.71073)	MoKα (λ = 0.71073)
2Θ range for data collection/°	9.738 to 134.994	6.646 to 51.992	6.708 to 51.996
Index ranges	$-13 \leq h \leq 14$, $-9 \leq k \leq 9$, $-33 \leq l \leq 26$	$-11 \leq h \leq 11$, $-11 \leq k \leq 11$, $-20 \leq l \leq 19$	$-11 \leq h \leq 11$, $-8 \leq k \leq 11$, $-20 \leq l \leq 16$
Reflections collected	13994	8279	8296
Independent reflections	2413 [R_{int} = 0.0308, R_{sigma} = 0.0191]	2809 [R_{int} = 0.0182, R_{sigma} = 0.0193]	2854 [R_{int} = 0.0232, R_{sigma} = 0.0250]
Data/restraints/parameters	2413/0/190	2809/0/199	2854/0/199
Completeness to theta = 25.0°	99.2%	98.6%	1.004
Goodness-of-fit on F^2	1.001	1.001	98.1%
Final R indexes [I>=2σ (I)]	R_1 = 0.0278, wR_2 = 0.0670	R_1 = 0.0291, wR_2 = 0.0741	R_1 = 0.0229, wR_2 = 0.0614
Final R indexes [all data]	R_1 = 0.0310, wR_2 = 0.0690	R_1 = 0.0320, wR_2 = 0.0760	R_1 = 0.0268, wR_2 = 0.0635
Largest diff. peak/hole / e.$Å^{-3}$	0.24/-0.20	0.27/-0.31	0.35/-0.32

NMR spectra

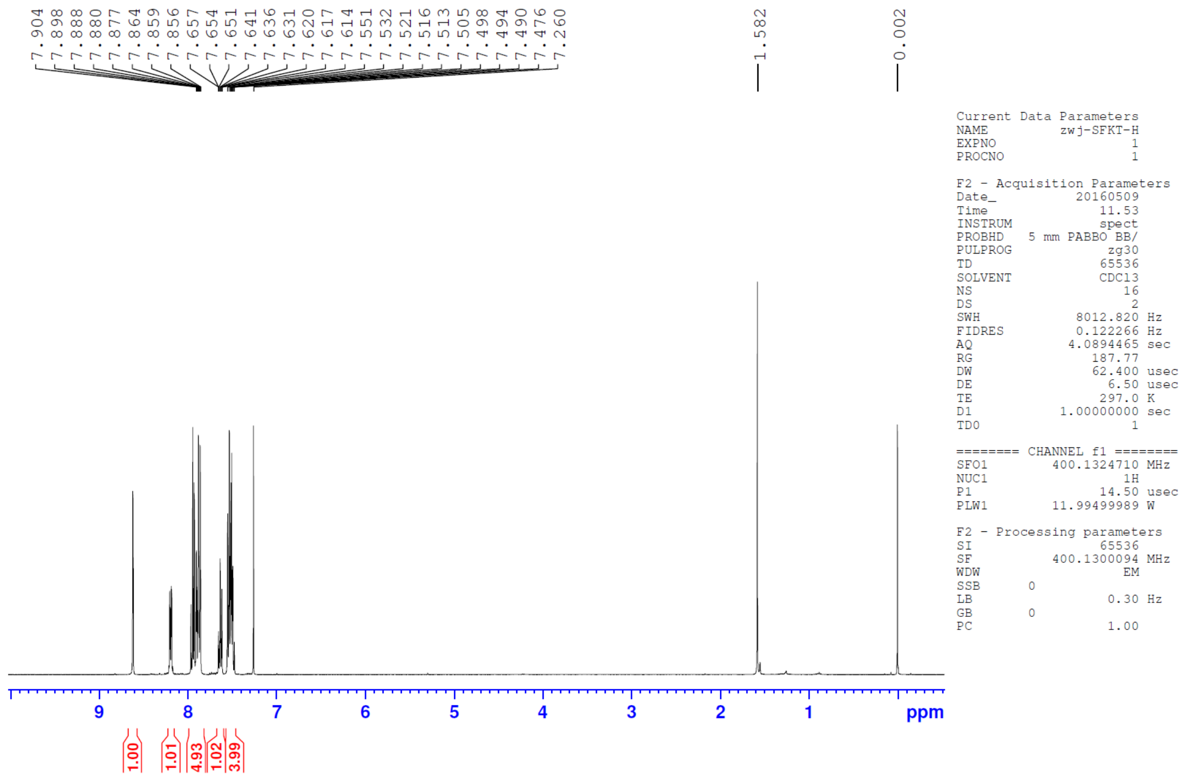

Supplementary Figure 21. ^{1}H NMR spectrum of **BDBT**.

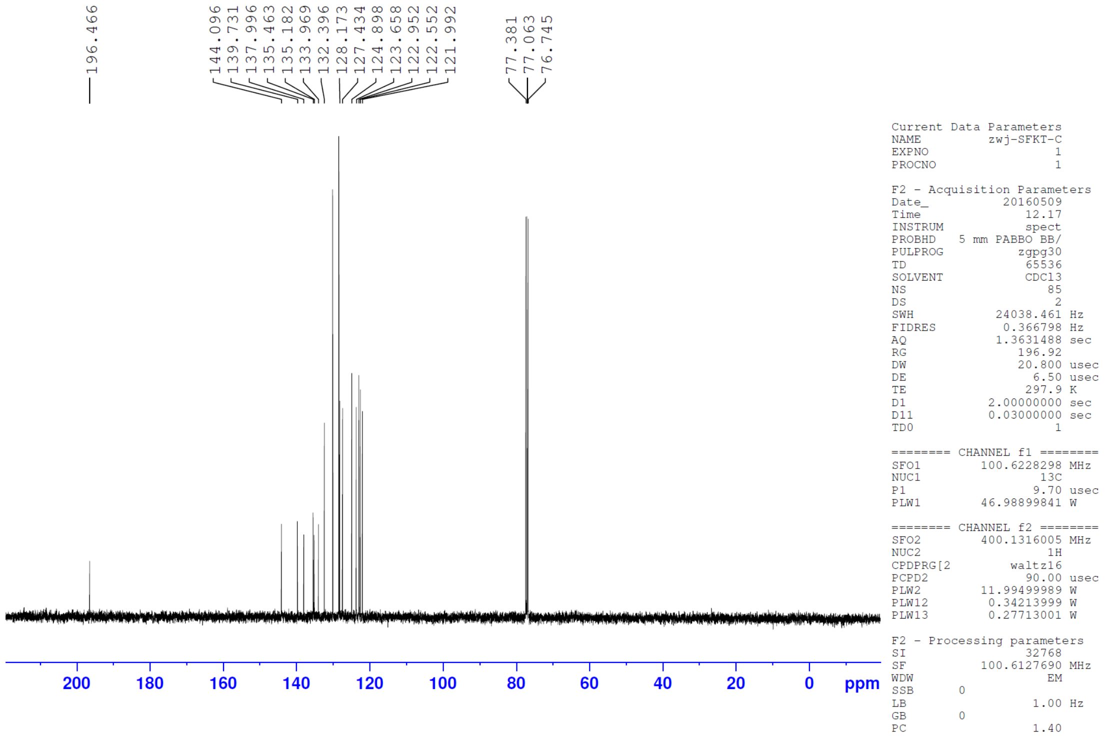

Supplementary Figure 22. ^{13}C NMR spectrum of **BDBT**.

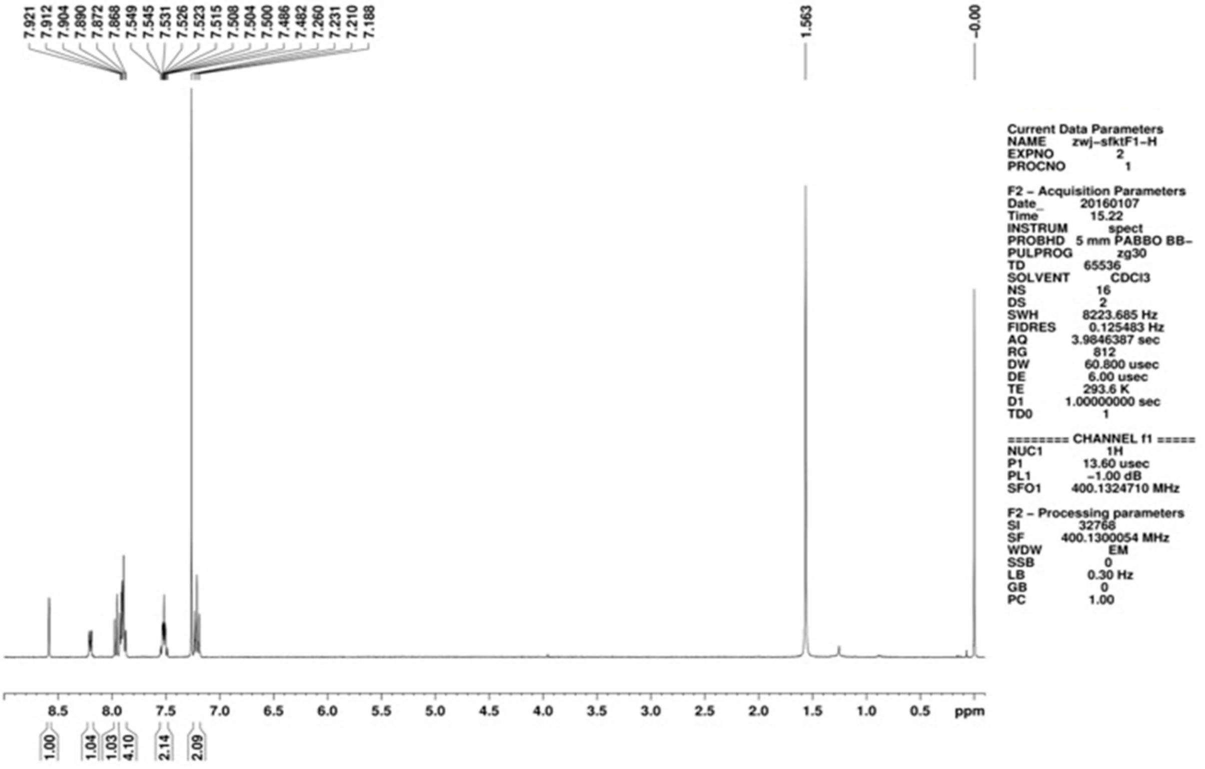

Supplementary Figure 23. ^{1}H NMR spectrum of **FBDBT**.

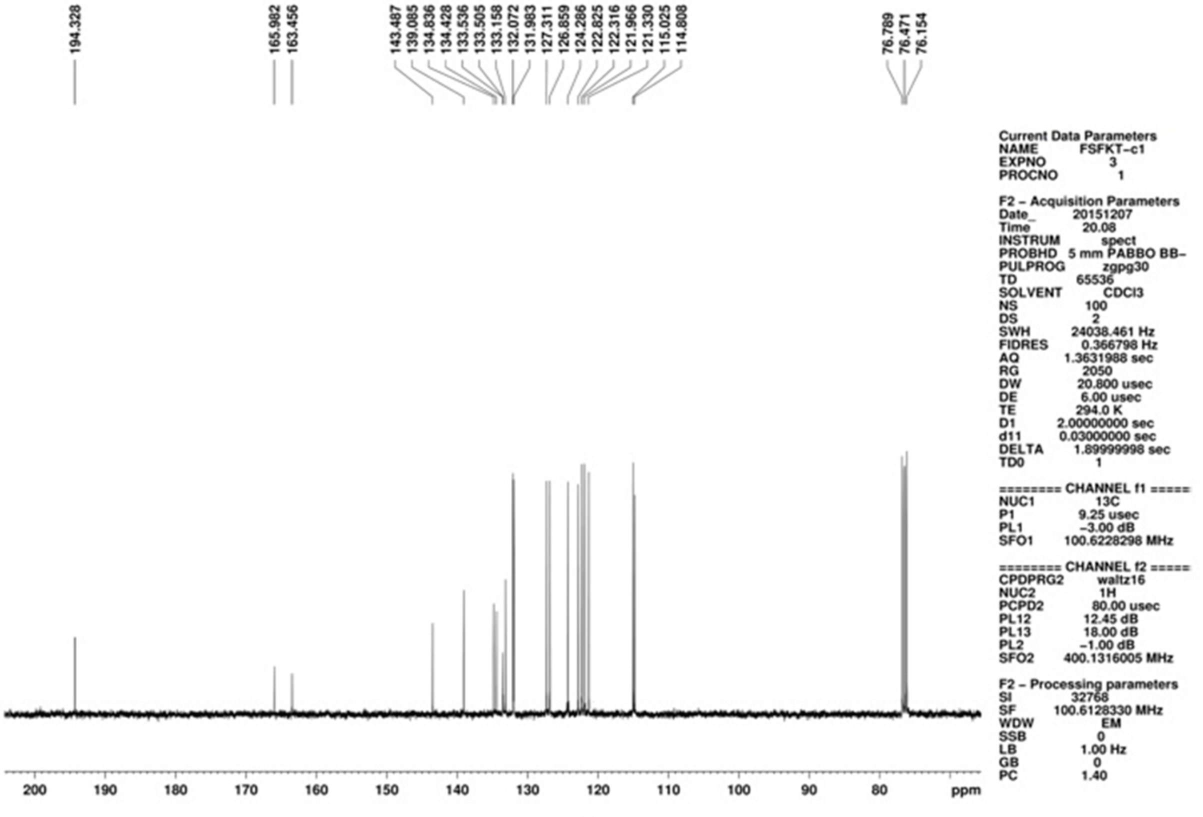

Supplementary Figure 24. ^{13}C NMR spectrum of **FBDBT**.

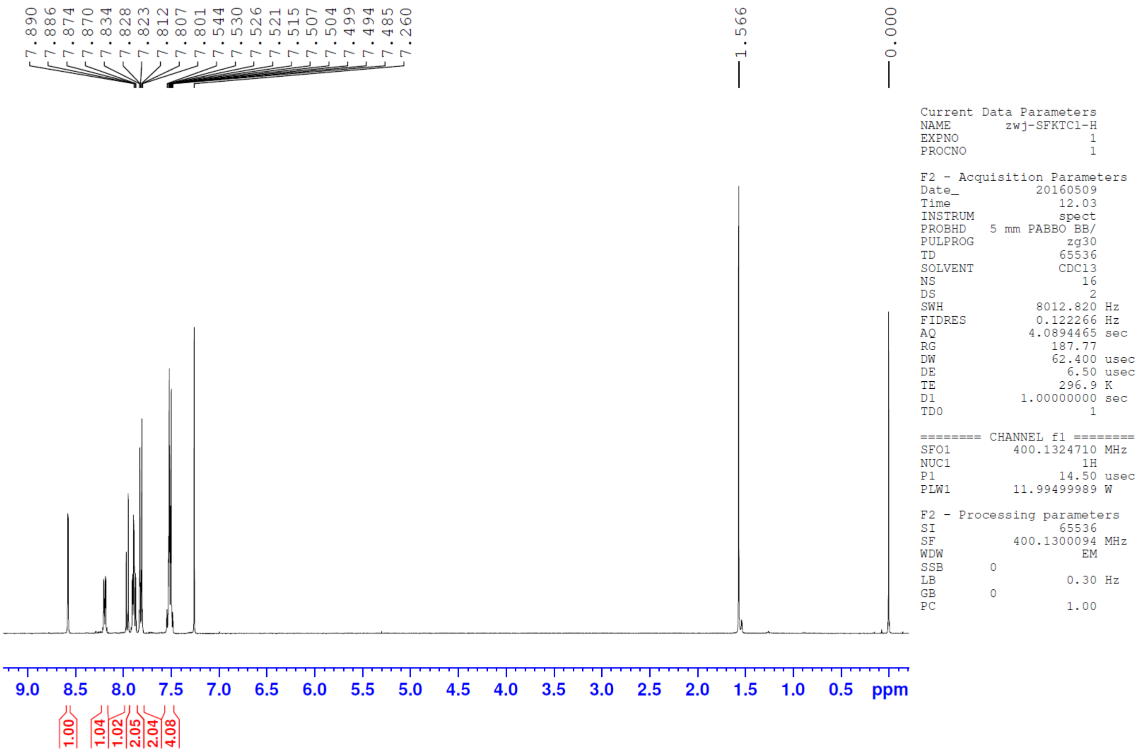

Supplementary Figure 25. ^{1}H NMR spectrum of **ClBDBT**.

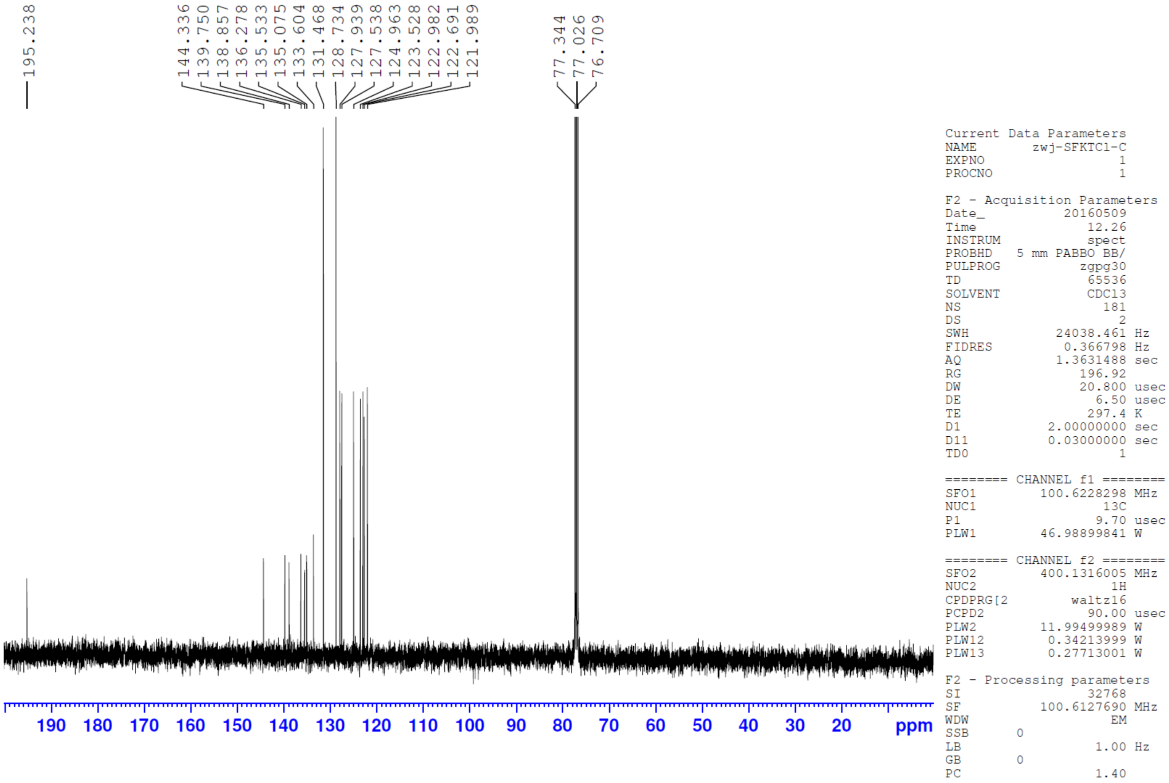

Supplementary Figure 26. ^{13}C NMR spectrum of **ClBDBT**.

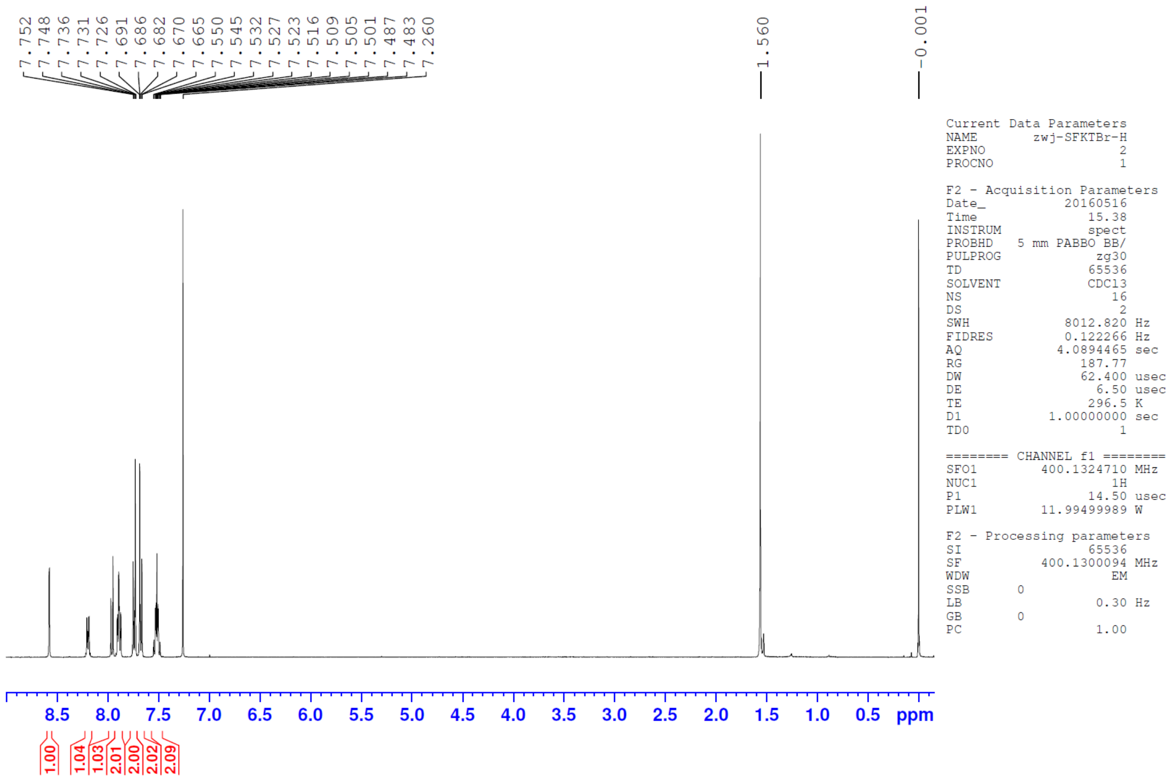

Supplementary Figure 27. ^{1}H NMR spectrum of **BrBDBT**.

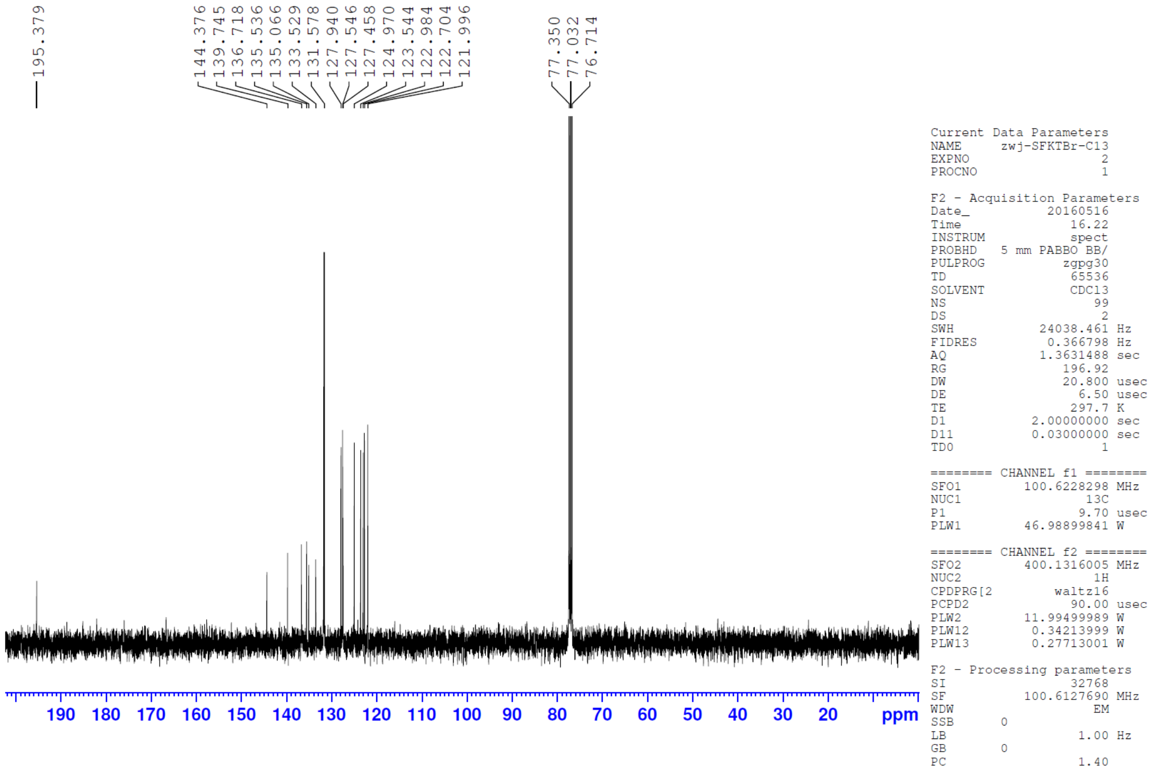

Supplementary Figure 28. ^{13}C NMR spectrum of **BrBDBT**.

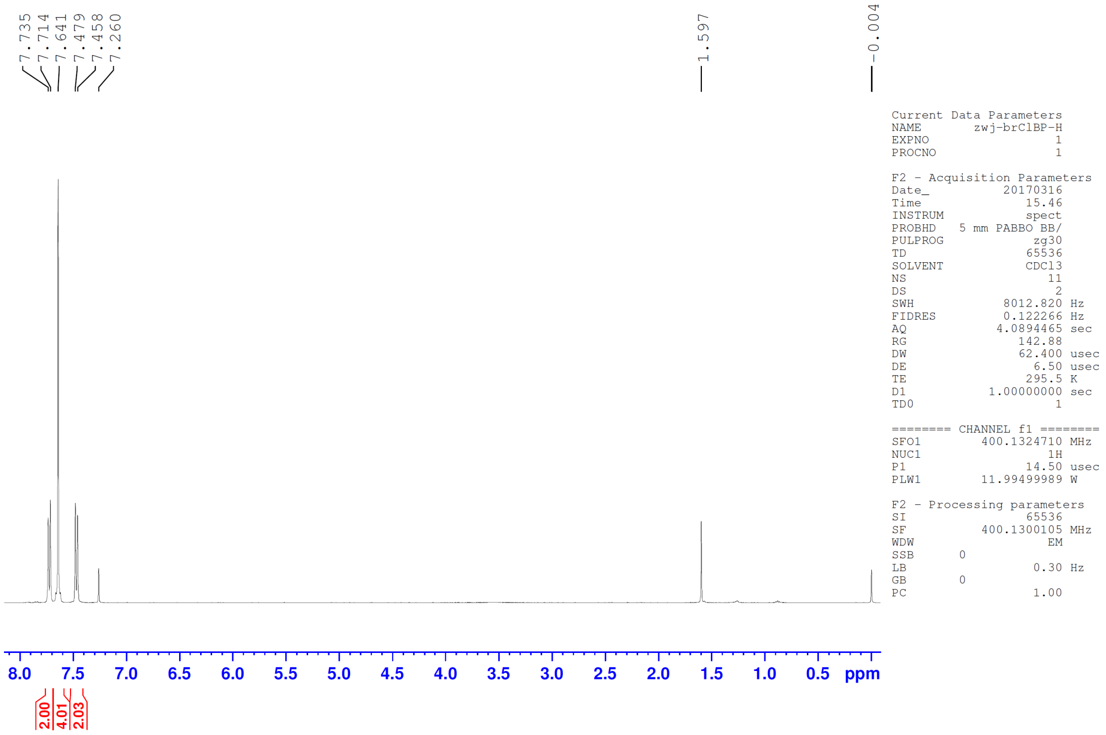

Supplementary Figure 29. ^{1}H NMR spectrum of **BCBP**.

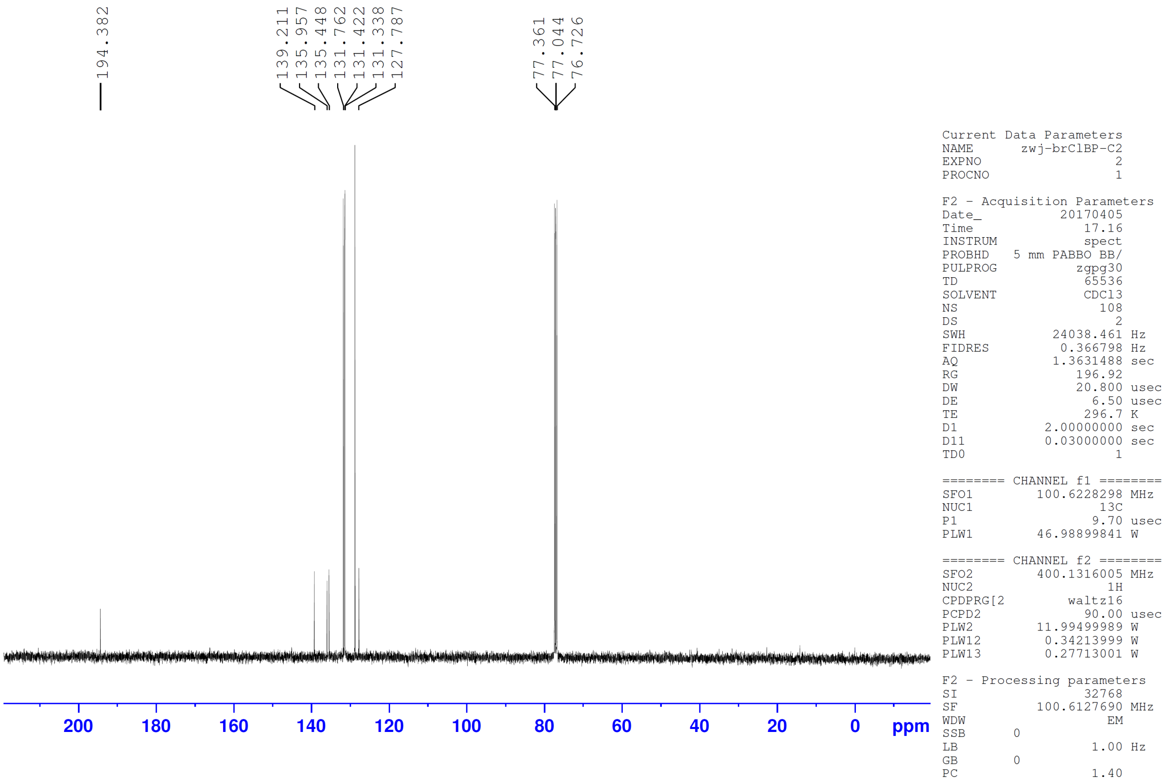

Supplementary Figure 30. ^{13}C NMR spectrum of **BCBP**.

Supplementary References

(1) Sherwood, P.; de Vries, A. H.; Guest, M. F.; Schreckenbach, G.; Catlow, R. C. A.; French, S. A.; Sokol, A. A.; Bromley, S. T.; Thiel, W.; Turner, A. J.; Billeter, S.; Terstegen, F.; Thiel, S.; Kendrick, J.; Rogers, S. C.; Casci, J.; Watson, M.; King, F.; Karlsen, E.; Sjøvoll, M.; Fahmi, A.; Schäfer, A.; Lennartz, C. QUASI: A General Purpose Implementation of the QM/MM Approach and Its Application to Problems in Catalysis. *J. Mol. Struct.: Theochem* **2003**, 632, 1-28.

(2) Ahlrichs, R.; Bär, M.; Häser, M.; Horn, H.; Kölmel, C. Electronic Structure Calculations on Workstation Computers: The Program System Turbomole. *Chem. Phys. Lett.* **1989**, 162, 165-169.

(3) Smith, W.; Forester, T. R. DL_POLY_2.0: A General-Purpose Parallel Molecular Dynamics Simulation Package. *J. Mol. Graph.* **1996**, 14, 136-141.

(4) Wang, J.; Wolf, R. M.; Caldwell, J. W.; Kollman, P. A.; Case, D. A. Development and Testing of a General Amber Force Field. *J. Comput. Chem.* **2004**, 25, 1157-1174.

(5) Bayly, C. I.; Cieplak, P.; Cornell, W.; Kollman, P. A. A Well-Behaved Electrostatic Potential Based Method Using Charge Restraints for Deriving Atomic Charges: The RESP Model. *J. Phys. Chem.* **1993**, 97, 10269-10280.

(6) Liu, W.; Wang, F.; Dai, D.; Li, L.; Dolg, M. The Beijing Four-Component Density Functional Program Package (BDF) And Its Application to EuO, EuS, YbO and YbS. *Theor. Chem. Acc.* **1997**, *96*, 75-83.

(7) Liu, W.; Hong, G.; Li, L. The Beijing Density Functional (BDF) Program Package: Methodologies and Applications, *J. Theor. Comput. Chem.* **2003**, *2*, 257-272.

(8) Hirao, K.; Ishikawa, Y. Recent Advances in Computational Chemistry, World Scientific, Singapore, **2004**, *5*, p257.

(9) Shuai, Z. G.; Peng, Q.; Niu, Y. L.; Geng, H. MOMAP, A Free and Open-Source Molecular Materials Property Prediction Package; Revision 0.2.004; Available Online: http://www.shuaigroup.net/; Shuai group: Beijing, CN, **2014**.

(10) Shuai, Z.; Peng, Q. Excited States Structure and Processes: Understanding Organic Light-Emitting Diodes at the Molecular Level. *Phys. Rep.* **2014**, 537, 123-156.
